# A 2024 inventory of test methods relevant to thyroid hormone system disruption for human health and environmental regulatory hazard assessment

**DOI:** 10.12688/openreseurope.18739.1

**Published:** 2024-11-06

**Authors:** Lucia Vergauwen, Lola Bajard, Sabrina Tait, Ingrid Langezaal, Anita Sosnowska, Alessandra Roncaglioni, Ellen Hessel, Annick D van den Brand, Ann-Cathrin Haigis, Jiří Novák, Klára Hilscherová, Natalia Buławska, Nafsika Papaioannou, Elisavet Renieri, Eliana Spilioti, Anastasia Spyropoulou, Arno C Gutleb, Henrik Holbech, Dimitra Nikolopoulou, Miriam N Jacobs, Dries Knapen

**Affiliations:** 1 Zebrafishlab, Veterinary Physiology and Biochemistry, University of Antwerp, Wilrijk, 2610, Belgium; 2 RECETOX, Faculty of Science, Masaryk University, Brno, 611 37, Czech Republic; 3 Center for Gender-Specific Medicine, Istituto Superiore di Sanità, Rome, 00161, Italy; 4 European Commission Joint Research Centre Ispra, Ispra, Lombardy, 21027, Italy; 5 Faculty of Chemistry, University of Gdansk, Gdańsk, 80-308, Poland; 6 Laboratory of Environmental Chemistry and Toxicology, Department of Environmental Health Sciences, Istituto di Ricerche Farmacologiche Mario Negri IRCCS, Milan, Lombardy, 20156, Italy; 7 National Institute for Public Health and the Environment, Bilthoven, Utrecht, 3721, The Netherlands; 8 HERACLES Research Center on the Exposome and Health, Center for Interdisciplinary Research and Innovation, Aristotle University of Thessaloniki, Thessaloniki, 570 01, Greece; 9 Laboratory of Toxicological Control of Pesticides, Scientific Directorate of Pesticides’ Control and Phytopharmacy, Benaki Phytopathological Institute, Athens, Attica, 145 61, Greece; 10 Environmental Sustainability Assessment and Circularity (SUSTAIN) Unit, Luxembourg Institute of Science and Technology, Belvaux, 4422, Luxembourg; 11 Department of Biology, University of Southern Denmark, Odense, 5230, Denmark; 12 Radiation, Chemical and Environmental Hazards, Harwell Innovation Campus, UK Health Security Agency, Chilton, OX11 0RQ, UK

**Keywords:** Thyroid hormone system disruption, endocrine disruption, new approach methods, One Health

## Abstract

Thyroid hormone system disruption (THSD) is a growing concern in chemical hazard assessment due to its impact on human and environmental health and the scarce methods available for assessing the THSD potential of chemicals. In particular, the general lack of validated in silico and *in vitro* methods for assessing THS activity is of high concern. This manuscript provides an inventory of test methods relevant to THSD. Building on the Organisation for Economic Co-operation and Development (OECD) Guidance Document 150 and recent international developments, we highlight progress in in silico and *in vitro* methods, as well as *in vivo* assays. The provided inventory categorizes available methods according to the levels of the OECD Conceptual Framework, with an assessment of the validation status of each method. At Level 1, 12 in silico models that have been statistically validated and are directly related to THSD have been identified. At Level 2, 67 *in vitro* methods have been listed including those assessed in key initiatives such as the European Union Network of Laboratories for the Validation of Alternative Methods (EU-NETVAL) validation study to identify potential thyroid disruptors. At Levels 3-5, THSD-sensitive endpoints are being included in existing fish-based OECD Test Guidelines to complement amphibian assays. In total, the inventory counts 108 entries comprising established methods (e.g., OECD Test Guidelines) as well as citable methods that are under further development and in some cases are ready for validation or in the initial stages of validation. This work aims to support the ongoing development of strategies for regulatory hazard assessment, such as integrated approaches to testing and assessment (IATAs), for endocrine disruptors, addressing critical gaps in the current testing landscape for THSD in both human and environmental health contexts.

## Plain language summary

Endocrine disruption - the potential of chemicals, such as industrial chemicals or pesticides, to disrupt hormonal systems and cause adverse health effects - is of growing concern due to its impact on human and environmental health and the scarce methods available for assessing such hazards. In particular, the limited methods available for assessing disruption of the thyroid hormone system, is of high concern. This manuscript provides an inventory of test methods relevant for the assessment of thyroid hormone system disruption. We highlight progress in different types of methods such as computer simulations, cell-based methods, non-mammalian embryo-based methods and animal methods and include an assessment of the readiness of each method for implementation in chemical evaluations. In total, the inventory counts 108 entries comprising already established methods as well as recent developments. This work aims to support the ongoing development of strategies for evaluating endocrine disruption, addressing critical gaps in the current testing landscape for thyroid hormone system disruption in both human and environmental health contexts.

## Introduction

The hazard assessment of chemicals with respect to endocrine disruption has been, and continues to be, of high regulatory priority in Europe and internationally. Over 70 years ago wildlife sentinel effects of endocrine perturbation by industrial, pesticidal and pharmaceutical chemicals started to be reported, with population level effects. Later, associations with adverse health impacts in humans also began to be noted, with well documented multigeneration effects in the instance of diethylstilbestrol (DES, a nonsteroidal oestrogen medication) use, for example. The reported effects were operating on oestrogenic and androgenic axes, and an extensive body of scientific evidence documents this. Based on this evidence, regulatory tools to assess the hazards of potentially (anti)oestrogenic and (anti)androgenic chemicals were developed and agreed at international governmental levels under the Organisation for Economic Co-operation and Development (OECD) Mutual Acceptance of Data (MAD) agreement. Up until the late 1990s, and into the start of the early 2000s, these oestrogenic and androgenic endpoints were only assessed using *in vivo* rodent Test Guidelines (TGs), such as the uterotrophic and Hershberger assays (TG 440 and 441 respectively), and the 28 and 90-day repeated dose chronic toxicity TGs (TG 407 and 408, respectively). From 2016, some endpoint measurements specific to thyroid hormone system disruption (THSD) were included in the standard *in vivo* rodent TGs for assessing human health effects. This includes mandatory assessment of the thyroid gland (e.g., organ weight and histopathological investigation) and mandatory or optional measurement of thyroid hormones (thyroxine [T4], triiodothyronine [T3] and/or thyroid-stimulating hormone [TSH]) in some TGs (summarized in Melching Kollmuss *et al.* (2023)). For assessing THS-specific activity and effects of chemicals in the environment, amphibian-based TGs have become available since 2009. These include the amphibian metamorphosis assay (AMA, TG 231), the larval amphibian growth and development assay (LAGDA, TG 241) and the *Xenopus* embryonic thyroid signalling assay (XETA, TG 248).

In 2012, the OECD published the first version of its Guidance Document on Standardised Test Guidelines for Evaluating Chemicals for Endocrine Disruption (Guidance Document [GD] 150), to provide guidance for the screening and testing of endocrine disrupting chemicals in a regulatory context ( OECD, 2012), and it was updated in 2018 ( OECD, 2018a). This guidance provides an overview of methods and assays for evaluating chemicals that had been validated or were under validation to become TGs, at the time of publication. The GD organizes these methods into five different levels according to the OECD Conceptual Framework (CF) for testing and assessment of endocrine disrupting chemicals. Level 1 test methods involve existing data and existing or new non-test information, including quantitative structure-activity relationships (QSARs) and other in silico/machine learning prediction tools. Level 2 and Level 3 assays are *in vitro* and *in vivo* assays, respectively, providing data about selected endocrine mechanisms or pathways (i.e., endocrine activity). Levels 4 and 5 are *in vivo* assays providing data on adverse effects on endocrine-relevant endpoints, with Level 5 assays providing more comprehensive data over more extensive parts of the life cycle of the organism ( OECD, 2018a). The list of methods provided in GD 150, including their organization into the different CF levels, constitutes the current basis of the ECHA-EFSA Guidance for the identification of endocrine disruptors ( ECHA-EFSA, 2018).

A number of critical gaps have been identified in the list of available, validated ^1^ (standardised) *in vitro* and *in vivo* methods for assessing thyroid hormone system disrupting chemicals (THSDCs). First, although *in vitro* test method development for estrogenic, androgenic and steroidogenic activity at Level 2 of the OECD CF started to enter validation in the early 2000’s with TG adoption from 2009 onwards, validated Level 2 assays for THSD are still completely lacking today. In that context, an OECD Scoping Document for THSD *in vitro* and *ex vivo* assays for the identification of modulators of thyroid hormone signalling was first published in 2012 and revised in 2014 ( Murk *et al.*, 2013; OECD, 2014b), with a call from the OECD soon after requesting OECD member countries to address the development and validation of relevant *in vitro* THSD methods. Various efforts followed internationally at the European Union (EU) and United States Environmental Protection Agency (US EPA) levels in particular. The Joint Research Centre (JRC)'s EU Reference Laboratory for alternatives to animal testing (EURL ECVAM) initiated and coordinated a validation study with its European Union Network of Laboratories for the Validation of Alternative Methods (EU-NETVAL), to assess the performance of methods that were considered to be relevant for THSDC identification ( Bernasconi *et al.*, 2023). Furthermore, many *in vitro* methods for assessing THSDCs have been developed or are under development in the context of EU research programmes such as in several of the EURION cluster projects (Especially the EURION projects focused on THSD: ERGO: Holbech *et al.*, 2020, ATHENA: Kortenkamp *et al.*, 2020, and SCREENED: Moroni *et al.*, 2020, summarized in the EURION methods table: Audouze *et al.*, 2024), and in the Partnership for the Assessment of Risks from Chemicals (PARC) ( Ramhoj *et al.*, 2023a). In the US, the US Environmental Protection Agency (US EPA) implemented the ToxCast/Tox21 program to screen chemicals with *in vitro* high-throughput assays, including those assessing THSD within the framework of the Endocrine Disruptor Screening Program (EDSP) ( Noyes *et al.*, 2019; Wang *et al.*, 2018; Wang *et al.*, 2019; Wang *et al.*, 2021). These are not validated methods, but data generated using ToxCast/Tox21 assays are freely available ( https://comptox.epa.gov/dashboard). Similarly, Level 1 in silico methods for assessing THSDCs are currently lacking in the OECD CF ( OECD, 2018a), but many are available and further development is ongoing. These do not undergo the validation process as described for *in vitro* and *in vivo* methods ( Jacobs *et al.*, 2024; OECD, 2005), but should ideally meet the reporting needs documented in the relevant OECD guidance. The last 15+ years of experience in regulatory applications of (Q)SARs and related in silico tools has highlighted that not all predictions produced by a valid ( OECD, 2014c, first published in 2007) model are acceptable for all regulatory purposes ( OECD, 2023). Finally, for environmental THSDC assessment, only amphibian tests are currently available. Several of the existing fish TGs measure rather general, apical endpoints that are sensitive to, but not diagnostic of, the T modality (such as growth and reproduction, ( OECD, 2018a)), but validated fish-based assays measuring endpoints that are more specific for the thyroid modality are currently lacking. To fill this gap, the addition of four THSD-sensitive endpoints to the Fish Early Life Stage Toxicity test (FELS test, TG 210) and the Fish Embryo Acute Toxicity test (FET test, TG 236) is currently under OECD validation as part of project 2.64 of the OECD Test Guidelines work plan (Inclusion of thyroid endpoints in OECD fish Test Guidelines). These augmented fish TGs are intended to complement the amphibian assays and overall give a broader picture of THSD in lower vertebrates.

Because of the great international interest in THSD assessment methods, and given the major developments that have taken place in the field since the last update of GD 150, here we provide a collated overview table of available THSD test methods including their validation status, to date. The methods are sorted into separate tables according to the different OECD CF Levels, and where relevant and possible we distinguish between methods for assessing human and environmental health. Nevertheless, it should be emphasised that the THS is well conserved among vertebrates and human health methods could be informative for environmental health assessment and vice versa ( Haigis *et al.*, 2023; LaLone *et al.*, 2018). For consistency and ease of reference, the Level 2 assays have been grouped into eight assay blocks corresponding to the OECD THSD Scoping Document ( OECD, 2014b): Central regulation, thyroid hormone synthesis, binding and transport in serum, metabolism and excretion, local cellular concentrations, cellular responses, short-term assays integrating multiple modes of action, and integrative cellular assays.

## Overview of available THSD methods

The following sections provide a brief commentary on the methods listed for each Level of the CF highlighting the most important advances that have been made compared to GD 150 ( OECD, 2018a). The tables include already validated methods, including those already used in regulatory frameworks, as the background. Additional methods that have been developed but not (yet) validated, or that are under validation, have been included. Since the main goal of this effort is to provide an overview of available methods with a regulatory implementation perspective, methods that are in the initial stages of development and which could not be cited yet, have not been included. For each method, we included a general appreciation of the test method readiness/validation status. For Level 2–5 assays, we distinguish between A - validated (TG), B - optimized, ready to be (pre-)validated/ validation ongoing, C - not validated, D - validation unsuccessful. This categorization was performed in alignment with, but not strictly according to, the agreed criteria developed in the OECD thyroid scoping document ( OECD, 2014b). Further details are provided where relevant.

### Level 1: Existing data and existing or new non-test information

For this effort, we focused on the availability of in silico models to predict THSD-relevant mechanisms. In silico models include (Q)SARs that, depending upon the quantity of the data used to build and test the model, can be constructed at the global (large chemical applicability domain) or local (chemical class type chemical domain) level, three dimensional and multidimensional molecular modelling, machine learning, neural networks and other more complex statistical tools including Bayesian approaches. Because our effort aims to provide an overview of methods with regulatory application in mind, we mainly searched for methods available in open-source predictive tools (e.g. Vega hub, Danish (Q)SAR Database or Endocrine Disruptome Tool) and only report models that have been statistically validated and for which documentation and/or a citation could be provided. Models that are directly related to THSD are listed in Table 1 and are grouped according to the blocks defined in the OECD THSD Scoping Document ( OECD, 2014b). For two core blocks (thyroid hormone synthesis, and cellular responses) ( Table 1) 12 in silico models in total have been identified, but in silico tools were not identified for the other blocks. Additionally, for hepatic nuclear receptor activation (Extended Data 1 - Table S1) 14 in silico models have been identified. Additional statistically validated models for THSD may be available in the literature. To explore this potential, we performed a targeted literature search (Extended Data 2). In total, 358 papers related to the subject were found and could be further analysed to find additional sufficiently validated methods to predict THSD-relevant mechanisms. Such analysis was not part of the scope of the present effort.

**Table 1.** Level 1 methods for THSD evaluation. List of in silico methods for THSD evaluation that fit into Level 1 (Existing data and existing or new non-test information) of the OECD Conceptual Framework for Testing and Assessment of Endocrine Disrupting Chemicals ( OECD, 2018a). Methods have been categorized according to the blocks introduced in the OECD THSD scoping document ( OECD, 2014b).

Method title	THSD mechanism / effect	Short method description	Test method readiness/ validation status *Regulatory acceptance, Statistically validated, Under development and evaluation*	Availability	Reference or link
BLOCK #2: THYROID HORMONE SYNTHESIS					
TPO inhibition QSAR1	TPO inhibition	A categorical QSAR model based on structural features and numeric molecular descriptors. (only LEADSCOPE model)	Statistically validated	DQdb	https://qsardb.food.dtu.dk/db/index.html https://qsarmodels.food.dtu.dk/download/qmrf/LS.TP1.pdf
TPO inhibition QSAR2	TPO inhibition	A categorical QSAR model based on structural features and numeric molecular descriptors. (only LEADSCOPE model)	Statistically validated	DQdb	https://qsardb.food.dtu.dk/db/index.html https://qsarmodels.food.dtu.dk/download/qmrf/LS.TP2.pdf
TPO inhibition Amplex Ultra Red (AUR) assay (TPO inhibition OBERON ^2^ model)	TPO inhibition	A categorical (binary) QSAR model based on k-nn algorithm and Dragon descriptors for AUR-TPO assay data	Statistically validated	VEGA	( Gadaleta *et al.*, 2021)
Sodium/iodine symporter (NIS), higher sensitivity	NIS inhibition	A categorical QSAR model to predict NIS inhibition. (only LEADSCOPE model)	Statistically validated	DQdb	https://qsardb.food.dtu.dk/db/index.html
BLOCK #6 CELLULAR RESPONSES					
Thyroid Receptor alpha effect (Nuclear Receptor-mediated Endocrine Activity, NRMEA)	TRα agonism and antagonism	This model is a hierarchical tree on three levels: fragments on first and second levels define if a compound is active on the receptor, third fragments define the type of activity of the compounds on the receptor.	Statistically validated	VEGA	https://www.vegahub.eu/vegahub-dwn/qmrf/QMRF_TRa_NRMEA.pdf
TR alpha Binding (IC50 in mg/l) (human in vitro)	TRα agonism and antagonism	Continuous (Q)SAR model made by use of Partial Least Squares (PLS) regression method. (Model is a consensus of three models, LEADSCOPE, Muticase ULTRA and SciMatics SciQSAR).	Statistically validated	DQdb	https://qsardb.food.dtu.dk/db/index.html https://qsardb.food.dtu.dk/download/qmrf/SQ_TRA.pdf
Thyroid Receptor beta effect (NRMEA)	TRβ agonism and antagonism	Model is a hierarchical tree on three levels: fragments on first and second levels define if a compound is active on receptor, third fragments define the type of activity of the compounds on the receptor	Statistically validated	VEGA	https://www.vegahub.eu/vegahub-dwn/qmrf/QMRF_TRb_NRMEA.pdf
Docking analysis - results of docking score [kcal/mol] to specific ligand - TRα (PDB: 3ILZ)	TRα agonism and antagonism	Endocrine Disruptome is an open source, user friendly and web-based prediction tool. It uses molecular docking to predict binding of compounds to receptors	Statistically validated	Endocrine disruptome	http://endocrinedisruptome.ki.si/ ( Kolšek *et al.*, 2014)
Docking analysis - results of docking score [kcal/mol] to specific ligand - TRβ (PDB: 3IMY)	TRβ agonism and antagonism	Endocrine Disruptome is an open source, user friendly and web-based prediction tool. It uses molecular docking to predict binding of compounds to receptors	Statistically validated	Endocrine disruptome	http://endocrinedisruptome.ki.si/ ( Kolšek *et al.*, 2014)
Binding score: compound - ligand TRα (PDB: 3ILZ) QSAR-MLR model	TRα agonism and antagonism	QSAR-MLR model for predicting the binding probability to the receptor based on simulated docking scores of 43 PFAS.	Statistically validated	Publication	( Kowalska *et al.*, 2023)
Binding score: compound - ligand TRβ (PDB: 3IMY) QSAR-MLR model	TRβ agonism and antagonism	QSAR-MLR model for predicting the binding probability to the receptor based on simulated docking scores of 43 PFAS.	Statistically validated	Publication	( Kowalska *et al.*, 2023)
TR beta Binding (IC50 in mg/l) (human *in vitro*)	TRβ agonism and antagonism	Continuous (Q)SAR model using a Partial Least Squares (PLS) regression method. (Model is a consensus of three models, LEADSCOPE, Muticase ULTRA and SciMatics SciQSAR).	Statistically validated	DQdb	https://qsardb.food.dtu.dk/db/index.html

^2^OBERON is one of the EURION cluster projects ( Audouze *et al.*, 2020)

In silico/machine learning methods can be used as independent methodology (utilising experimental data generated from the THSD assays) at Level 1 (existing information), but can also provide data and decision tree type analysis to support the development of test batteries or Integrated Approaches to Testing and Assessment (IATAs). They have advantages over the other non-animal methods – in chemico and *in vitro* – due to the time savings, cost reduction, environmental friendliness, but of course they do depend upon well-curated relevant experimental data in the first place to build and test the models. To date these models have not yet been put forward for addition to the OECD QSAR Toolbox, where there is some regulatory peer review.

### Level 2: *In vitro* methods providing data on THSD mechanisms

Level 2 assays have been categorized according to the blocks introduced in the OECD THSD scoping document ( OECD, 2014b) for easy reference. Additionally, in order to provide a broader perspective on mechanisms that are relevant – but not specific – to THSD, Extended Data 1 (Table S2) lists assays to study hepatic nuclear receptor activation (leading to increased liver clearance of THs and thus indirectly linked to Block 4 ‘metabolism and excretion’) as an example of how THSD can be studied in a broader context.

Based on the complete lack of validated *in vitro* methods for THSD, recently a lot of attention has been given to the development and validation of Level 2 methods. In 2017 the Joint Research Centre (JRC)'s EURL ECVAM selected 18 relevant assays based on the OECD scoping document ( OECD, 2014b) (indicated as ‘NETVAL method’ in Table 2) and invited EU-NETVAL laboratories to implement the methods, optimise them where necessary, develop Standard Operating Procedures (SOPs) and perform experimental studies to assess their reproducibility and reliability ( Bernasconi *et al.*, 2023). This resulted in a total of 8 methods that are, according to the OECD expert group for Thyroid Disruption Methods (OECD TDM EG), sufficiently optimised and ready to be transferred to other laboratories. These are given test method readiness/validation status B in Table 2, together with specific recommendations from the TDM EG for any further validation. A number of other citable and relevant methods that are under (further) development, or in some cases are ready for validation or in the initial stages of validation, have also been added to Table 2, but this list is not exhaustive. For example, this includes methods from EURION cluster projects ( Audouze *et al.*, 2024) and contract research organisations. We also included TH-related ToxCast/Tox21 assays (some of which have been recently reviewed in Forner-Piquer *et al.*, 2023), methods used to screen thousands of chemicals. These are given a regulatory test method readiness status C in Table 2. Although *in vitro* methods for THSD are also being developed within the PARC partnership (Project P5.2.1.c) as summarized by Ramhoj *et al.* (2023a), these assays were not included in Table 2 because of the generally early stage of method development. Taken together, important advances have been made and are continued to be made in filling the crucial gap of *in vitro* methods for THSD.

**Table 2.** Level 2 methods for THSD evaluation. List of methods for THSD evaluation that fit into Level 2 “ *In vitro* assays providing data about selected endocrine mechanism(s)/pathway(s) (mammalian and non-mammalian methods)” of the OECD Conceptual Framework for Testing and Assessment of Endocrine Disrupting Chemicals ( OECD, 2018a). Methods have been categorized according to the blocks introduced in the OECD THSD scoping document ( OECD, 2014b).

Method title	THSD mechanism / effect	Short method description	Test method readiness/ validation status *A - validated (TG)* *B - optimized, ready to be (pre-)validated/ validation ongoing* *C - not validated* *D - validation unsuccessful*	Reference or link
BLOCK #1 CENTRAL REGULATION				
NETVAL Method 1a PATHHUNTER® BETA-ARRESTIN thyrotropin-releasing hormone (TRH) receptor activation (beta-galactosidase) measuring agonist and antagonist activities	TRH receptor agonism and antagonism	This method is a chemiluminescent *in vitro* method from Eurofins-DiscoverX which measures the agonist and antagonist activities of test items by assessing the thyrotropin-releasing hormone receptor (TRHR) activation. The method employs the commercially available Chinese hamster ovary cells (CHO-K1) engineered to stably express the tagged proteins TRHR and β-Arrestin (PathHunter® CHO-K1 TRHR β-Arrestin Cell Line). Following chemical-TRHR binding, the ProLink™ tagged GPCR is activated inducing the recruitment of Enzyme Acceptor tagged β-Arrestin, forcing complementation of the two enzyme fragments. The resulting functional enzyme hydrolyses the substrate to generate a chemiluminescent signal.	C - no validation pending	TM2019-02 (EU) https://www.discoverx.com/technologies-platforms/enzyme-fragment-complementation-technology/cell-based-efc-assays/protein-protein-interactions/gpcrs-b-arrestin
Toxcast assays TOX21_TRHR_HEK293_Agonist TOX21_TRHR_HEK293_Antagonist	TRH receptor agonism and antagonism	This method is a cell-based assay, single-readout assay that uses HEK293T, a human kidney cell line. It has the potential to screen G protein-coupled receptors. A compound/drug can either activate (agonist) or suppress (antagonist) GPCRs and this can be monitored by measuring levels of intracellular calcium, as detected with Fluorescence intensity signals.	C	https://comptox.epa.gov/dashboard/assay-endpoints/TOX21_TRHR_HEK293_agonist https://comptox.epa.gov/dashboard/assay-endpoints/TOX21_TRHR_HEK293_antagonist
NETVAL Method 1b Thyrotropin-stimulating hormone (TSH) receptor activation based on cAMP measurement	TSH receptor agonism	This method measures the production of cAMP after activation of the thyroid stimulating hormone receptor (TSHR). The production of intracellular adenosine 3’,5’ cyclic monophosphate (cAMP) is important for thyroid hormone physiology. cAMP is a critical component of a signal transduction pathway linking TSHR and its ligand TSH to the activation of adenylate cyclase and therefore cAMP production. The method measures the intracellular cAMP concentration in Chinese hamster ovary cells (CHO-K1) transfected with the gene of the human TSHR, using an Enzyme Linked Immuno Sorbent Assay.	B - C - Assessed by the OECD TDM EG. Full assessment could not be done due to lack of active chemicals. Recommendation for further validation: Optimise the protocol, include antagonist mode of action, identify active chemicals and generate more data.	TM2019-03 (EU) ( Santini *et al.*, 2003)
Toxcast assays TOX21_TSHR_ HTRF_Agonist_ratio TOX21_TSHR_HTRF_Antagonist_ratio TOX21_TSHR_wt_Agonist_HTRF_ratio	TSH receptor agonism and antagonism	This method is a cell-based single-readout assay that uses HEK293T, a human embryonic kidney cell line. It is designed to make measurements of TSHR agonism or antagonism with cAMP used as indicator, detected with fluorescence intensity signals.	C	https://comptox.epa.gov/dashboard/assay-endpoints/TOX21_TSHR_HTRF_Agonist_ratio https://comptox.epa.gov/dashboard/assay-endpoints/TOX21_TSHR_HTRF_Antagonist_ratio https://comptox.epa.gov/dashboard/assay-endpoints/TOX21_TSHR_HTRF_Antagonist_ratio
BLOCK #2: THYROID HORMONE SYNTHESIS				
NETVAL Method 2a Thyroperoxidase (TPO) inhibition based on oxidation of Amplex UltraRed	TPO inhibition	This *in vitro* method evaluates the peroxidase activity of TPO, a key enzyme for the synthesis of thyroid hormones. The method allows to identify TPO-inhibiting test items by assessing the enzyme activity in prepared extracts from FTC-238 human follicular thyroid carcinoma cells transfected with human recombinant TPO. TPO activity is measured through the oxidation of a commercially-available peroxidase substrate (Amplex UltraRed, AUR, ® LifeTech) and its conversion to a fluorescent product.	B - Assessed by the OECD TDM EG. Recommendation for further validation: Update and confirm the SOP, then transfer the method to other laboratories to continue validation.	TM2019-04 (EU) Based on: ( Paul *et al.*, 2014; Paul Friedman *et al.*, 2016)
Toxcast assays CCTE_GLTED_hTPO TPO inhibition using Amplex Ultrared ( ERGO project)	TPO inhibition	This cell-free *in vitro* method evaluates the peroxidase activity of TPO using cell lysates from transfected HEK293 (Toxcast), HEK293T (ERGO) or Lenti-X ^TM^ ( Dong *et al.*, 2024) cells overexpressing hTPO with AUR for detection.	C - Comparable to NETVAL method 2a undergoing validation	https://comptox.epa.gov/dashboard/assay-endpoints/CCTE_GLTED_hTPO ( Dong *et al.*, 2020; Dong *et al.*, 2024) ERGO: ( Liu *et al.*, 2024)
Toxcast assays CCTE_Simmons_AUR_TPO Thyroid peroxidase inhibition assay on thyroid microsomes (Kaly-Cell ^3^)	TPO inhibition	This *in vitro* method evaluates the peroxidase activity of TPO in using rat and/or human thyroid microsomes with AUR for detection.	C - SOP and data have been provided to the OECD TDM EG, for assessment and recommendations on further validation. The TDM EG commented that human thyroid microsomes are a challenge for world-wide applicability of a TG.	( Paul *et al.*, 2014; Paul Friedman *et al.*, 2016) https://comptox.epa.gov/dashboard/assay-endpoints/CCTE_Simmons_AUR_TPO Kaly-Cell: Asselin *et al.* (unpublished report)
NETVAL Method 2c Tyrosine iodination using liquid chromatography	TPO inhibition	This *in vitro* method provides information on the TPO-mediated iodination of tyrosine. The conversion from L-tyrosine to Iodotyrosine by TPO that is present in prepared extracts from FTC-238 human follicular thyroid carcinoma cells transfected with human recombinant TPO, is measured with LC-MS/MS.	B - Assessed by the OECD TDM EG. Recommendation for further validation: Ready for transfer to other laboratories to continue validation.	TM2019-06 (EU) ( Divi & Doerge, 1996; Doerge & Takazawa, 1990; Freyberger & Ahr, 2006; Reinen *et al.*, 2024; Tater *et al.*, 2021)
TPO inhibition using ICP-MS ( ERGO project)	TPO inhibition	This *in vitro* method evaluates the effect on both peroxidation and iodide organification activities of TPO. It uses ICP-MS to measure the conversion of L-tyrosine to Iodotyrosine by TPO in cell lysates from transfected HEK293T cells overexpressing hTPO.	C - Comparable to NETVAL method 2c undergoing pre-validation	Liu *et al.* (unpublished report) based on ( Liu *et al.*, 2024)
NETVAL Method 2b TPO inhibition using luminol Same method used by other developers with different cell lines.	TPO inhibition	This *in vitro* method evaluates the peroxidase activity of TPO. Luminescence is used to measure peroxidase activity in cell lysates from transfected HEK293T or FTC-238 cells overexpressing hTPO.	D - validation stopped. The method was found to be unspecific to TPO ( Liu *et al.*, 2024)	TM2019-05 (EU) ( Jomaa *et al.*, 2015; Liu *et al.*, 2024)
Toxcast Assay CCTE_Simmons_GUA_TPO	TPO inhibition	This *in vitro* method evaluates the peroxidase activity of TPO using the guaiacol oxidation assay with pig thyroid gland tissue as the source of TPO.	C	( Paul *et al.*, 2014; Tietge *et al.*, 2013)
TPO inhibition using guaiacol oxidation assay ( Concept Life Sciences ^4^)	TPO inhibition	This *in vitro* method evaluates the peroxidase activity of TPO. It uses the guaiacol oxidation assay. The test method is available for isolated human/rat/pig/dog thyroid microsomes.	C - Developed to GLP standardization	Studies available on request from CLS. Not yet in the public domain: Rat: CLS4_0001_0019 Dog: CLS4_0001_0030 Pig: CLS4_0001_0031 Human: CLS4_0001_0032
NETVAL Method 2d Inhibition of the sodium iodide symporter (NIS) based on Sandell-Kolthoff reaction	NIS inhibition	This *in vitro* method measures the active transport of extracellular iodide across the cellular membrane of Mel624-CMV hNIS-NEO human melanoma cells expressing a functional human NIS, an intrinsic membrane glycoprotein that actively transports iodide from blood into thyroid follicular cells during the initial step of TH synthesis. This method is designed to identify test items that inhibit NIS-mediated iodide uptake by directly competing with iodide for intracellular transport, or indirectly, through an inhibition of Na+/K+ ATPase that disrupts maintenance of the critical cross-membrane sodium gradient required for NIS functionality. Reduction of intracellular iodide is measured as optical density with a spectrophotometric readout following the Sandell-Kolthoff (SK) reaction. The SK-reaction provides a colorimetric indicator of iodine concentration through the reaction between quadrivalent cerium and trivalent arsenic catalyzed by free iodide in sulphuric acid solution.	C – TDM EG assessment is pending availability of report and data. A limited amount of data could be produced due to discontinuation of the cell line. Further validation efforts with a new cell line will be needed.	TM2019-07 (EU) ( Hallinger *et al.*, 2017; Waltz *et al.*, 2010; Wang *et al.*, 2018)
Inhibition Potential of Rat or Human sodium iodide symporter (NIS) function in recombinant HEK cells ( Kaly-Cell) Human NIS inhibition overexpressed in recombinant HEK cells ( ERGO project) NIS inhibition in Fischer Rat Thyroid Low-Serum 5% (FRTL-5) cells. ( Concept Life Sciences)	NIS inhibition	This *in vitro* method provides information on the potential to inhibit the active transport of extracellular iodide across the cellular membrane. It employs the SK reaction on the HEK293 cell line that overexpresses the human or rat NIS or FRTL-5 cells.	B - C – Kaly-Cell method SOP and data have been provided to the OECD TDM EG, for assessment and recommendations on further validation .	Kaly-Cell: Asselin *et al.* (unpublished report) ERGO: Kumari *et al.* (unpublished results) Study reports available on request from CLS. Study No. Validation study: CLS4_0040_0010
Na+/I- symporter (NIS) inhibition human NIS transfected in MCF7 cells	NIS inhibition	This *in vitro* method provides information on the potential to inhibit the active transport of extracellular iodide across the cellular membrane. It employs the SK reaction on the MCF7hNIS breast cancer cell line that overexpresses the human sodium iodine symporter (NIS). Similar results have been observed with this cell line as compared to the HEK293 hNIS cell line from the US-EPA.	C	( Dong *et al.*, 2019)
Toxcast Assay CPHEA_Stoker_NIS_Inhibition_RAIU	NIS inhibition	This *in vitro* method uses hNIS-HEK293T-EPA, a novel human kidney cell line expressing human NIS. This radioactive iodide uptake (RAIU) assay is used to identify inhibitors of NIS-mediated iodide uptake.	C	( Wang *et al.*, 2021)
Human iodotyrosine deiodinase (IYD) inhibition assay (included in ATHENA project)	IYD inhibition	This method uses recombinant human IYD enzyme produced in a baculovirus system with insect cells. The assay measures IYD-liberated iodide with the SK reaction using the native full length form of IYD, monoiodotyrosine (MIT) as the substrate and NADPH as the reducing agent.	C	( Olker *et al.*, 2021; Olker *et al.*, 2022; Renko *et al.*, 2016; Shimizu *et al.*, 2013)
BLOCK #3: BINDING AND TRANSPORT IN SERUM				
NETVAL Method 3a Thyroxine-binding prealbumin (TTR) / thyroxine-binding globulin (TBG) binding using fluorescence displacement (ANSA) Toxcast Assay CCTE_GLTED_hTTR	TTR/TBG binding	This in chemico method investigates interference of test items with binding of thyroid hormones to human serum transport proteins 'thyroxine-binding prealbumin' (TTR) and 'thyroxine-binding globulin' (TBG). The binding of 8-anilino-1-naphtalenesulfonic acid (ANSA) to TTR or TBG produces fluorophore complexes with intrinsic fluorescence signal. In the presence of competitive ligands, displacement of ANSA occurs and the fluorescence signal detected decreases. Binding/displacement capacity of endocrine/thyroid disrupting chemicals can be assessed and compared to that of 3,3’,5-triiodo-L-thyronine (T3) and/or T4.	B - Assessed by the OECD TDM EG. Recommendations for further validation: Update and confirm the SOP, Include assessment of TBG, then transfer the method to other laboratories to continue validation.	TM2019-08 (EU) ( Cao *et al.*, 2010; Montano *et al.*, 2012)
NETVAL Method 3b Thyroxine-binding prealbumin (TTR) binding using fluorescence displacement (T4-FITC) Same method used in the ERGO project	TTR binding	This in chemico method investigates interference of test items with binding of thyroid hormones to human serum transport protein 'thyroxine-binding prealbumin (TTR)'. The addition of TTR-binding compounds to a mixture of TTR and a fluorescent conjugate FITC-T4 causes a decrease in fluorescence, because the TTR-binding compounds compete FITC-T4 out of the TTR binding-pocket and the FITC-T4 conjugate exhibits higher fluorescence intensity when bound to TTR than in the unbound condition.	B - Assessed by the OECD TDM EG. An SPSF was submitted by PEPPER and accepted onto the OECD work programme. Validation continued by PEPPER.	TM2019-09 (EU) ( Hamers *et al.*, 2020)
TTR binding using mass spectrometry	TTR binding	A triple bioaffinity MS (BioMS) concept is presented in which recombinant TTR (rTTR) and MS are used for different analytical objectives, i.e., rapid screening, confirmation, and identification. First, a rapid and easy to use radiolabel-free competitive MS binding assay uses a stable isotope of a model analyte (13C6-T4) to screen indirectly for the affinity toward rTTR. The isotope-labeled competitor is measured by ultrahigh performance-liquid chromatography-electrospray ionizationtriple-quadrupole-MS (UPLC-QqQ-MS), operating in a dedicated single reaction monitoring mode (SRM). The amount of measured label is indicative for the amount and affinity of rTTR-active compounds in the sample. Second, the same rTTR biorecognition element was used in a bioaffinity isolation procedure in combination with UPLC-QqQ-MS but operating in a multiple reaction monitoring mode (MRM). Third, for identification of any unknown ligands having rTTR bioaffinity, the same rTTR biorecognition element was used in a bioaffinity isolation procedure in combination with UPLC-QTOF-MS operating in the high resolution full-scan accurate mass mode.	C	( Aqai *et al.*, 2012)
TTR binding using radioactivity	TTR binding	This assay measures the release of (free) radiolabelled T4 (125I-T4) from hTTR in the presence of waste water treatment plant extracts or the positive control, 2,2',3,4',5,5',6-heptachloro-4-biphenylol (4-OH-CB187).	C	( Meerts *et al.*, 2000; Ucan-Marin *et al.*, 2010)
TTR Binding assay	TTR Binding	Free T4, potential competitor chemical and TTR are incubated and the unbound T4 and free chemical molecules are separated from TTR complexes using BioGel columns.	C	( Collet *et al.*, 2020)
BLOCK #4 METABOLISM AND EXCRETION				
NETVAL Method 4a Deiodinase 1 activity based on Sandell-Kolthoff reaction	DIO1 inhibition	This method assesses the inhibition of thyroid hormone deiodination, the major pathway regulating thyroid hormone bioavailability in mammalian tissues. The method measures the activity of the deiodinase (DIO) 1 enzyme in human liver microsomes (≥ 25 donors of mixed gender and various age). The uninhibited enzyme liberates free iodide from the substrate. Free iodide is measured using the SK method.	B - Assessed by the OECD TDM EG. An SPSF was submitted by PEPPER and accepted onto the OECD work programme. Validation continued by PEPPER.	TM2019-10 (EU)
ToxCast Assays CCTE_GLTED_hDIO1, CCTE_GLTED_hDIO2, CCTE_GLTED_hDIO3	DIO1, DIO2, DIO3 inhibition	These assays are designed to test the inhibitory activity of chemicals toward human DIO1, DIO2 or DIO3 enzymes using HEK293 cells transiently transfected with hDIO1, hDIO2 or hDIO3. The uninhibited enzyme liberates free iodide from the substrate. Free iodide is measured using the SK method.	C	https://comptox.epa.gov/dashboard/assay-endpoints/CCTE_GLTED_hDIO1 https://comptox.epa.gov/dashboard/assay-endpoints/CCTE_GLTED_hDIO2 https://comptox.epa.gov/dashboard/assay-endpoints/CCTE_GLTED_hDIO3 ( Hornung *et al.*, 2018; Olker *et al.*, 2019)
Human and rat deiodinase inhibition assay (Deiodinase 1, 2 and 3, Kaly-Cell)	DIO1, DIO2, DIO3 inhibition	*In vitro* inhibition assay of rat and human DIO1, DIO2 and DIO3 using recombinant HEK293 cells ectopically expressing rat or human DIO1, DIO2 or DIO3. For the assay, T4 is used as the substrate for the three DIOs and the deiodination of T4 to T3 (by DIO1 and DIO2) or of T4 to reverse T3 (rT3, 3,3'5'-triodo-L-thyronine, by DIO3) is determined by measuring T3 or rT3 levels via High Performance Liquid Chromatography-High Resolution Mass Spectrometry (HPLC-HRMS).	B - C - SOP and data have been provided to the OECD TDM EG, for assessment and recommendations on further validation	Asselin *et al.* (unpublished report)
Human, rat and dog deiodinase inhibition assay (Deiodinase 1, 2 and 3) ( Concept Life Sciences)	DIO1, DIO2, DIO3 inhibition	DIO activities are determined by conversion of T4 or rT3 substrate to T3 (or rT3) and 3,3’-diiodo-L-thyronine (3,3’-T2). The method uses an extract of HEK293 cells expressing recombinant human/rat/dog DIO1, DIO2 and DIO3. Reaction products are quantified by LC-MS/MS.	C-B - GLP standardization is underway.	Study reports available from CLS at year end 2024
Deiodinase 1 activity based on mass spectrometry	DIO1 inhibition	Mass spectrometry–based method for measuring the activity of DIOs in human liver microsomes (DIO1 activity). Although outer ring deiodination (ORD) is catalysed by both DIO1 and DIO2, these reactions presumably only involve DIO1 because it has been shown that DIO2 is not expressed in human liver ( Richard *et al.*, 1998) but is highly expressed in other tissues such as the brain, pituitary, and brown adipose tissue. Instrumental analysis was performed by LC with positive electrospray MS/MS (LC-MS/MS) using conditions modified from ( Wang & Stapleton, 2010).	C	( Butt *et al.*, 2011; Wang & Stapleton, 2010)
Deiodinase 1, 2 and 3 inhibition assay ( ERGO project, ATHENA project)	DIO1, DIO2, DIO3 inhibition	DIO1, 2, 3 activities are determined by assessment of free iodide cleaved from rT3, T4, and T3 substrates, respectively. The iodide is assessed using a spectrophotometric absorbance readout following the SK reaction. Based on Renko *et al.* (2015). DIO activities are assessed in cell lysates of transfected HEK293T cells overexpressing human DIO1, DIO2 and DIO3.	C - Comparable to NETVAL method 4a, based on DIO1 in human microsomes.	( Renko *et al.*, 2012; Renko *et al.*, 2015; Weber *et al.*, 2022) ERGO: Nešporová *et al.* (unpublished results)
Deiodinase 1 inhibition assay ( ERGO project)	DIO1 inhibition	DIO1 activity is determined by assessment of free iodide cleaved from rT3 substrate. The iodide is assessed using a spectrophotometric absorbance readout following the SK reaction. Based on Renko *et al.* (2015). DIO1 activity is assessed in cell lysates of HepG2 cells intrinsically expressing DIO1.	C - Comparable to NETVAL method 4a, based on DIO1 in human microsomes.	( Renko *et al.*, 2015) ERGO: Nešporová *et al.* (unpublished results)
Deiodination activity measurement using radioimmunoassay (RIA) and chromatography on Sephadex LH-20 columns	DIO1 inhibition	This method measures DIO activity with RIA in human liver microsomes using RIA and chromatography on Sephadex LH-20 columns.	C	( Visser *et al.*, 1988)
NETVAL Method 4b Inhibition of thyroid hormones (THs) glucuronidation using liquid chromatography/mass spectrometry (LC/MS)	Glucuronidation inhibition	This method assesses the potential of test items to inhibit the metabolism and excretion of THs. Glucuronidation is one of the main pathways in the metabolism of THs (T3 and T4). The method consists in the incubation of either T4 or T3 with human liver microsomes (from ≥ 50 donors of mixed gender and various ages) that contain UDP-glucuronosyltransferases (UGT), the enzyme involved in the glucuronidation. Following separation and purification, the mixture is analysed by liquid chromatography/mass spectrometry (LC/MS). Acyl- and Phenolic- Glucuronides are separated and peak identities confirmed.	B – Assessed by the OECD TDM EG. Recommendation for further validation: Generate more data on inactive chemicals to confirm specificity. Transfer to other laboratories to continue validation.	TM2019-11 (EU) ( Tong *et al.*, 2007)
Toxcast assays CLD_UGT1A1_6hr, 24hr or 48hr	Glucuronidation inhibition	This method is one of 16 assay component(s) measured or calculated from the CellzDirect (CLD) assay. It is designed to make measurements of mRNA induction, a form of inducible reporter, as detected with chemiluminescence signals by Quantitative Nuclease Protection Assay (qNPA) technology. It is a cell-based, multiplexed-readout assay that uses hepatocytes, human liver primary cells, with measurements taken at 6, 24 or 48 hours after chemical dosing.	C	https://comptox.epa.gov/dashboard/assay-endpoints/CLD_UGT1A1_6hr https://comptox.epa.gov/dashboard/assay-endpoints/CLD_UGT1A1_24hr https://comptox.epa.gov/dashboard/assay-endpoints/CLD_UGT1A1_48hr ( Rotroff *et al.*, 2010)
Toxcast assays LTEA_HepaRG_UGT1A1 LTEA_HepaRG_UGT1A6	Glucuronidation inhibition	The assay is based on a high-throughput transcriptomic analysis of 93 transcripts related to xenobiotic receptor activation, including UGT1A1 and UGT1A6 in human liver HepaRG cells treated for 48h. Gene expression is evaluated by qRT-PCR.	C	https://comptox.epa.gov/dashboard/assay-endpoints/LTEA_HepaRG_UGT1A1 https://comptox.epa.gov/dashboard/assay-endpoints/LTEA_HepaRG_UGT1A6 ( Franzosa *et al.*, 2021)
NETVAL Method 4c Inhibition of thyroid hormones (THs) sulphation using liquid chromatography.	Sulphation inhibition	This method assesses the potential of test items to inhibit the metabolism and excretion of THs. Sulphation is one of the main pathways in the metabolism of THs (T3 and T4). The method consists in the incubation of either T4 or T3 with human liver cytosol of mixed gender donors sulphotransferase (SULT), the enzyme involved in the sulphation. Following separation and purification, the mixture is analysed by liquid chromatography.	C	TM2019-12 (EU)
Toxcast assays CLD_SULT2A_6hr, 24hr or 48hr	Sulphation inhibition	This method is one of 16 assay component(s) measured or calculated from the CLD assay. It is designed to make measurements of mRNA induction of sulphotransferase (SULT) as detected with chemiluminescence signals by qNPA technology. It is a cell-based, multiplexed-readout assay that uses hepatocytes, human liver primary cells, with measurements taken at 6, 24 or 48 hours after chemical dosing.	C	https://comptox.epa.gov/dashboard/assay-endpoints/CLD_SULT2A_6hr https://comptox.epa.gov/dashboard/assay-endpoints/CLD_SULT2A_24hr https://comptox.epa.gov/dashboard/assay-endpoints/CLD_SULT2A_48hr ( Rotroff *et al.*, 2010)
Toxcast assays LTEA_HepaRG_SULT2A1	Sulphation inhibition	The assay is based on a high-throughput transcriptomic analysis of 93 transcripts related to xenobiotic receptor activation, including SULT2A1. Human liver HepaRG cells are treated for 48h. Gene expression is evaluated by qRT-PCR.	C	https://comptox.epa.gov/dashboard/assay-endpoints/LTEA_HepaRG_SULT2A1 ( Franzosa *et al.*, 2021)
Induction of thyroxine (T4) glucuronidation in primary hepatocytes ( Kaly-Cell)	Glucuronidation induction	This method assesses the induction of T4 glucuronide in rat and human primary hepatocytes cultured in a 2D-sandwich configuration with a 7-day exposure. T4, T4-G, T4-S and T3/rT3 are quantified using HPLC-HRMS.T4 metabolism was 9 times higher in primary rat hepatocytes than in primary human hepatocytes.	C This method was validated in-house	( Parmentier *et al.*, 2022; Baze *et al.*, 2024)
UGT Induction assay utilizing LC-MS/MS ( Concept Life Sciences)	Glucuronidation induction	This method is designed for: a) Assessment of induction of CYP (Cytochrome P450) and UGT genes by measuring mRNA levels. b) T4-UGT Enzyme activity Assay: Assessment of enzyme activity by LC-MS/MS bioanalysis of formation of T4-Glucuronide metabolite and T4 clearance in rat and human hepatocyte incubates. Primary Rat and Human Hepatocytes are treated up to 6 days in culture. CYP and UGT mRNA levels are measured and a T4 UGT enzyme activity assay is conducted based on LC-MS/MS. Measurement of T4 Clearance is optional.	C-B - Developed to GLP standardization	Study available on request to CLS CLS4_0026_0020 Poster presentation at SOT and EUROTOX (refs to be added)
UDPGT activity measurement using radioactivity and chromatography on Sephadex LH-20 mini-columns	Glucuronidation induction	Radioactivity measurements of T3 and T4 UGT activities in rat liver microsomes. Hepatic T3 and T4 UGT activities were quantified as the amount of 125I-T4 or 125I-T3 glucuronide produced (separated by chromatography). Developed by Beetstra *et al.* (1991) and modified by Hood & Klaassen (2000).	C	( Beetstra *et al.*, 1991; Hood & Klaassen 2000)
Sulphotransferase activity measurement using radioactivity and chromatography on Sephadex LH-20 mini-columns	Sulphation induction	Assessment of iodothyronine sulphotransferase activities after incubation of T4, T3, rT3 or 3,3'-T2 and ^125^I-labeled compound with liver cytosol or recombinant sulphotransferase preparations in the presence or absence of 3’-phosphoadenosine-5’phos-phosulphate (PAPS). 3,3'-T2 sulphate formation was analysed by chromatography on Sephadex LH-20 minicolumns as described by Visser *et al.*, (1993). Method was used for preliminary characterization of iodothyronine sulfotransferase activities of rat and human liver cytosol and recombinant rSULT1C1 and hSULT1A1 isoenzymes.	C	( Beetstra *et al.*, 1991; Visser *et al.*, 1993; Visser *et al.*, 1998)
Sulphotransferase activity measurement using radioactivity and SPE LC-MS/MS	Sulphation induction/inhibition	Modification of method by Visser *et al.* (1998). The primary modifications are the use of human liver cytosol and solid phase extraction (SPE) for sample clean-up and the use of LC-MS/MS for thyroid hormones and sulphated metabolite analysis.	C	( Butt & Stapleton, 2013)
Oestrogen sulphotransferase (SULT 1E1) activity and supplies of PAPS synthetase as a cofactor	Sulphation induction/inhibition	The sulphation process relies on the SULT enzymes and on supplies of PAPS as co-factor. For SULT 1E1 the methodology utilises human liver cytosol, prepared from snap-frozen human donor liver surplus to surgical requirements as the enzyme source of SULT 1E1 ( Harris *et al.*, 2005). Oestradiol (E2) sulphonation was measured using a method based on cDNA transfection ( Qian *et al.*, 1998). The quantiﬁcation of PAPSS1 mRNA expression uses the human neuronal medulloblastoma (TE671) cell line treated with test compounds for 24 h. Levels of mRNA were measured via quantitative real time PCR ( Turan *et al.*, 2005).	C-B	( Harris *et al.*, 2005; Qian *et al.*, 1998; Turan *et al.*, 2005; Waring *et al.*, 2012)
Sulphotransferase activity measurement using radioactivity with liquid scintillation spectrometry	Sulphation induction/inhibition	This method examined activity of the SULTs involved in iodothyronine metabolism during human placental development. SULT enzyme activity was measured with either PAP ^35^S or radioactively labelled substrates (iodothyronines) as described by Visser *et al.* (1998) and was optimized with respect to substrate, PAPS and cytosolic protein concentration, buffer composition, and incubation time.	C	( Stanley *et al.*, 2001)
TM2009-14 (EU) Cytochrome P450 (CYP) enzyme induction *in vitro* method using cryopreserved differentiated human HepaRG™ cells	Phase I induction	The human cytochrome P450 (CYP) enzyme induction method assesses the potential of test chemicals to induce the activity of three CYP enzymes (CYP1A2, CYP2B6 and CYP3A sub-family) in cryopreserved differentiated human HepaRG™ cells. At the molecular level, CYP enzyme induction is a process controlled by a set of nuclear receptors associated with downstream signal transduction pathways. The human HepaRG™ cell model is a suitable candidate human hepatic metabolic competent test system. A cocktail of three specific CYP enzymatic substrate probes is applied to the cells and CYP enzyme induction is assessed through the measurement of the specific metabolite formation by CYP1A2, 2B6 and 3A sub-family by LC/MS.	A – Validation finalised, chemical augmentation for proficiency chemicals finalised. An OECD Test Guideline is under development and additional testing in three laboratories, of chemicals to widen the applicability of the TG, is completed.	TM2009-14 (EU) ( Bernasconi *et al.*, 2019; Jacobs *et al.*, 2022) and additional manuscripts in prep.
Additional tools are available looking at enzyme induction using primary liver tissues, microsomes, S9 mix, .. (see below). However, during a validation exercise led by EURL ECVAM, the high variability of the primary human liver tissues led to the OECD WNT conclusion that this test method was insufficiently reproducible for test guideline purposes. The use of S9 mix as a shorter term application, pending validation of a more optimum method such as the CYP Induction test method ( Bernasconi *et al.*, 2019), was first reviewed in OECD in 2008 and published again in 2014 ( Jacobs *et al.*, 2008; Jacobs *et al.*, 2013; OECD, 2014a, first published in 2008).				
Cytochrome P450 (CYP) enzyme induction *in vitro* method (EROD) using fluorescence spectroscopy.	Phase I induction	Measurement of EROD (marker of CYP1A1 activity) in rat liver microsomes with fluorescence spectroscopy ( Burke & Mayer, 1974) and modifications to increase substrate stability ( Pohl & Fouts, 1980), increase throughput ( De Vito *et al.*, 1993).	C	( Burke & Mayer, 1974; De Vito *et al.*, 1993; Pohl & Fouts, 1980)
Cytochrome P450 (CYP) enzyme induction *in vitro* method (EROD) using fluorescence spectroscopy.	Phase I induction	Measurement of EROD activity (marker of CYP1A2 activity) in human and rat liver microsomes or cell monolayers using a fluorimetric assay.	C	( Burke *et al.*, 1985; Pearce *et al.*, 1996; Richert *et al.*, 2009)
Cytochrome P450 (CYP) enzyme induction *in vitro* method (acetanilide-4-hydroxylase assay) using reverse phase HPLC.	Phase I induction	Measurement of CYP1A2 activity (acetanilide-4-hydroxylase assay) in human and rat liver microsomes. Reverse phase HPLC-based assay.	C	( Liu *et al.*, 1991)
Cytochrome P450 (CYP) enzyme induction *in vitro* method (bupropion hydroxylase assay)	Phase I induction	Measurement of CYP2B6 activity (bupropion hydroxylation) in human and rat liver microsomes.	C	( Faucette *et al.*, 2000)
Cytochrome P450 (CYP) enzyme induction *in vitro* method (testosterone 6β-hydroxylase assay) using HPLC.	Phase I induction	Measurement of CYP3A4/5 activity (testosterone 6β-hydroxylation) in human and rat liver microsomes or cell monolayers. Most commonly an HPLC-based assay.	C	( Alexandre *et al.*, 2012; McKillop *et al.*, 1998; Richert *et al.*, 2009; Sonderfan *et al.*, 1987)
Cytochrome P450 (CYP) enzyme induction *in vitro* method (EROD, MROD) using HPLC with fluorescence detection	Phase I induction	Sensitive method for the determination of CYP1A activities such as EROD and MROD in liver microsomes from human, monkey, rat and mouse by high-performance liquid chromatography with fluorescence detection.	C	( Hanioka *et al.*, 2000)
BLOCK #5 LOCAL CELLULAR CONCENTRATIONS				
NETVAL Method 5a Inhibition of monocarboxylate transporter 8 (MCT8) based on Sandell-Kolthoff reaction Similar method in the ATHENA and PARC projects	TH membrane transporter inhibition	This *in vitro* method measures the active transport of T3 across the plasma membrane by monocarboxylate transporter 8 (MCT8). T3 uptake by Madin Darby Canine Kidney (MDCK) cells stably transfected with human MCT8, is measured with a specrophotometric absorbance readout following the SK reaction.	B - C Assessment by TDM EG is pending availability of the SOP, study report and data.	TM2019-13 (EU) ( Dong & Wade, 2017; Jayarama-Naidu *et al.*, 2015; Johannes *et al.*, 2016; Wagenaars *et al.*, 2024b)
Inhibition of thyroid hormone transmembrane transporter protein OATP1C1 ( ATHENA project)	TH membrane transporter inhibition	This *in vitro* method uses CHO-K1 cells overexpressing OATP1C1, and measures the uptake of radiolabeled T4 compared to control cell lines.	C - Info from Method Developer 09-2024: The SOP is in preparation. Optimisation of the protocol towards an LC-MS readout to avoid the use of radiolabelled T4 and facilitate transfer to another laboratory, is being considered.	( Wagenaars *et al.*, 2024a)
Inhibition of thyroid hormone transmembrane transporter protein OAT4 ( ATHENA project)	TH membrane transporter inhibition	This *in vitro* method uses MDCK-1 cells overexpressing OAT4, and measures the uptake of radiolabeled T4 compared to control cell lines.	C - Info from Method Developer 09-2024: The SOP is in preparation. Optimisation of the protocol towards an LC-MS readout to avoid the use of radiolabelled T4 and facilitate transfer to another laboratory, is being considered.	( Wagenaars *et al.*, 2024a)
BLOCK #6 CELLULAR RESPONSES				
NETVAL Method 6a Human thyroid hormone receptor alpha (TRα) and Human thyroid hormone receptor beta (TRβ) reporter gene transactivation measuring agonist activities	TR activation (agonism)	This method assesses the activation of thyroid hormone receptor alpha (TRα) and beta (TRβ). It is making use of proprietary human HEK293 cells engineered to provide constitutive, high-level expression of the human TRα and TRβ coupled with a highly responsive luciferase reporter gene (commercially available kit from INDIGO Biosciences). Quantification of changes in luciferase expression in the treated vs. untreated reporter cells by detection of luminescence provides a sensitive measure of changes in TR activation without collateral induction/suppression of genes that are otherwise regulated by these transcription factors. This method measures agonist TR functional activity.	B - Assessed by the OECD TDM EG. Recommendation for further validation: Include procedure to measure TR antagonism. Identify more active agonist and antagonist chemicals. Transfer to other laboratories for further validation.	TM2019-14 (EU) ( Robitaille *et al.*, 2022; Vanden Heuvel, 2021) https://indigobiosciences.com/wp-content/uploads/2022/04/TM_EUC-THR96LCMA-v7.2.pdf
NETVAL Method 6b CALUX human thyroid hormone receptor beta (TRβ) reporter gene transactivation measuring agonist and antagonist activities	TRβ activation (agonism and antagonism)	This method assesses the activation of TRβ using proprietary TRβ CALUX® cells which originated from a human osteoblastic osteosarcoma U2OS line. These cells express a functioning human TRβ coupled with a luciferase reporter gene. Test items with an agonist or antagonist effect on TR activity can be detected and an increase or decrease of TR signalling results in corresponding changes in the expression of luciferase activity that can be measured with a luminometer.	B – Assessed by the OECD TDM EG Recommendation for further validation: Identify more active agonist and antagonist chemicals. Transfer to other laboratories for further validation.	TM2019-15 (EU) ( Simon *et al.*, 2016)
Toxcast assays ATG_THRa1_TRANS ATG_THRb_TRANS2	TR activation (agonism)	These are cell-based, multiplexed-readout assays that use a HepG2 human liver cell line, with measurements taken at 24 h after chemical dosing. It is designed to make measurements of mRNA induction, as detected with fluorescence intensity signals by RT-PCR and capillary electrophoresis technology. Changes in fluorescence intensity signals are indicative of inducible changes in transcription factor activity. This is quantified by the level of mRNA reporter sequence unique to the transfected trans-acting reporter gene and exogenous transcription factors.	C	https://comptox.epa.gov/dashboard/assay-endpoints/ATG_THRa1_TRANS https://comptox.epa.gov/dashboard/assay-endpoints/ATG_THRb_TRANS2 ( Martin *et al.*, 2010; Medvedev *et al.*, 2018; Romanov *et al.*, 2008)
Toxcast assays ATG_hTRa_XSP1 and XSP2 ATG_hTRb_XSP1 and XSP2	TR activation (agonism)	ATG_XSP1/XSP2_multi-species_TRANS is a cell-based, multiplexed assay created by modifying the existing Attagene TRANS-FACTORIAL system to include a panel of nuclear receptors from some or all of the following species: human ( *Homo sapiens*), mouse ( *Mus musculus*), frog ( *Xenopus laevis*), zebrafish ( *Danio rerio*), chicken ( *Gallus gallus*), and turtle ( *Chrysemys picta*). The ECOTOX-FACTORIAL format uses HepG2, a human liver cell line, with measurements taken at 24 h after chemical dosing. Xsp1 includes 3 nM 6-alpha-fluorotestosterone to partially stimulate the androgen receptor. Xsp2 includes 3 nM 6-alpha-fluorotestosterone and 1.5 nM norgestrel to stimulate the androgen receptor. It is designed to make measurements of mRNA induction as detected with fluorescence intensity signals by RT-PCR and capillary electrophoresis technology. Changes in fluorescence intensity signals are indicative of inducible changes in transcription factor activity. This is quantified by the level of mRNA reporter sequence unique to the trans-acting reporter gene response element.	C	https://comptox.epa.gov/dashboard/assay-endpoints/ATG_hTRa_XSP1 https://comptox.epa.gov/dashboard/assay-endpoints/ATG_hTRa_XSP2 https://comptox.epa.gov/dashboard/assay-endpoints/ATG_hTRb_XSP1 https://comptox.epa.gov/dashboard/assay-endpoints/ATG_hTRb_XSP2 ( Medvedev *et al.*, 2020; Houck *et al.*, 2021)
Toxcast assays NVS_NR_hTRa_Antagonist	TRα activation (antagonism)	This method is a biochemical, single-readout assay that uses extracted gene-proteins in a cell-free assay. Measurements were taken 1 h after chemical dosing. It is an enzyme-linked immunosorbent assay with chemiluminescence signals by AlphaLISA immunoassay technology. Changes in chemiluminescence signals produced from the receptor-ligand binding of the key ligand T3 are indicative of a change in receptor antagonist activity for the human TRα.	C	https://comptox.epa.gov/dashboard/assay-endpoints/NVS_NR_hTRa_Antagonist ( Moriyama *et al.*, 2002; Knudsen *et al.*, 2011; Sipes *et al.*, 2013)
Toxcast assays TOX21_TR_LUC_GH3_Agonist TOX21_TR_LUC_GH3_Antagonist	TR activation (agonism and antagonism)	This method is a cell-based, single-readout assay that uses GH3, a rat pituitary gland cell line, with measurements taken at 28 h after chemical dosing. It is designed to make measurements of luciferase induction, as detected with bioluminescence signals by CellTiter-Glo Luciferase-coupled ATP quantitation technology. Changes in bioluminescence signals produced from an enzymatic reaction involving the key substrate [One-Glo] are indicative of changes in transcriptional gene expression due to agonist or antagonist activity regulated by the human TRα and TRβ.	C	https://comptox.epa.gov/dashboard/assay-endpoints/TOX21_TR_LUC_GH3_Agonist https://comptox.epa.gov/dashboard/assay-endpoints/TOX21_TR_LUC_GH3_Antagonist ( Freitas *et al.*, 2011; Freitas *et al.*, 2014; Paul-Friedman *et al.*, 2019)
TR transactivation	TR activation (agonism and antagonism)	A number of methods are based on transfection of different cell lines with plasmids carrying mostly the human TRα and/or TRβ genes coupled with a responsive luciferase reporter gene to provide information on the agonistic or antagonistic interaction with the TR. Cell lines used are: PZ-TR cells (based on HepG2 cells), CV-1 green monkey kidney cells, CHO-K1 hamster ovary cells, HeLa cells, HepG2 cells and PC12 rat adrenal cells. The latter is transfected with avian TR ( Valdehita *et al.*, 2014). Chemicals activating or inhibiting (in the presence of an activator) the receptor can be identified by changes in luminescence signals.	C	( Bai *et al.*, 2018; Du *et al.*, 2013; Fini *et al.*, 2012; Hu *et al.*, 2013; Ren *et al.*, 2019; Sharan *et al.*, 2014; Shi *et al.*, 2011; Shi *et al.*, 2012a; Shi *et al.*, 2012b; Shi *et al.*, 2016; Valdehita *et al.*, 2014; Illés *et al.*, 2015; Zhang *et al.*, 2011; Zhang *et al.*, 2016; Zhang *et al.*, 2019)
TR binding	TR activation (binding)	Different methods were used to measure the binding of chemicals to human TR, such as Surface plasmon resonance (SPR) or cell-free models using the rat or the human TRα1 Ligand Binding Domain (LBD), also in connection with recruitment of corepressor and coactivators.	C	( Lévy-Bimbot *et al.*, 2012; Nakamura *et al.*, 2013; Xiang *et al.*, 2017)
BLOCK #7 RELEVANT SHORT-TERM ASSAYS INTEGRATING MULTIPLE MOAS				
Human Thyroid Microtissue test method (US-EPA)	TH synthesis inhibition Altered T3 and T4 levels	Medium-throughput organotypic screening assay comprised of reconstructed human thyroid microtissues to quantitatively evaluate the disruptive effects of chemicals on T3 and T4 production and secretion. Assay endpoints include microtissue morphology, thyroid hormone synthesis, and viability. After stimulation with TSH, the following genes important for TH synthesis are expressed. TSHR, TG, TPO, SLC5A5 (NIS), SLC26A4 (pendrin), DUOX1, DUOX2, DUOXA1, DUOXA2.	B – validation ongoing	( Deisenroth *et al.*, 2020)
Mouse thyroid-on-a-Chip ( SCREENED project)	Nuclear receptor activation	This method uses organoids with mouse embryonic stem cells derived from thyroid follicles that retain expression of key thyroid genes and a typical follicular structure as well as T4 synthesis capacity. So far, transcriptional changes after exposure to a PAH have shown activation of the xenobiotic AhR pathway. Other responses remain to be investigated. As a further development of the system, a modular microfluidic organoid platform using LEGO-like bricks reduces operation time and allows the fluidic combination of various modular bricks with different functionalities. Complementing the current mouse thyroid-on-a-chip, a human thyroid-on-a-chip as well as bioprinted versions of the mice and human thyroid-on-a-chip are under development.	C	( Carvalho *et al.*, 2023; Carvalho *et al.*, 2024)
Thyroid gland explant culture assay	TH synthesis inhibition	*Ex vivo* assay based on *Xenopus laevis* thyroid gland explant culture in which inhibition of thyroxine (T4) release is the measured endpoint. Detection of the T4 released by the glands is done via commercially available canine T4 RIA kit using radioactive 125-I labelled T4. Although the assay may reflect the potential for a chemical to alter T4 release from the thyroid gland, the specific mechanism(s) by which this is produced are not identified.	C	( Hornung *et al.*, 2010; Hornung *et al.*, 2015)
BLOCK #8 INTEGRATIVE CELLULAR ASSAYS				
NETVAL Method 8a T-screen assay - Measurement of proliferation of rat pituitary-derived cell line GH3	TR activation (agonism and antagonism)	This method measures effects (agonism or antagonism) on the thyroid receptor via measurement of T3-dependent growth (induction or inhibition) in T3 depleted medium. The assay uses a rat pituitary tumour cell line (GH3) in serum-free medium. Compounds are tested in the absence and presence of 0.5 nM T3. The assay can be used to study interference of test items with TH at the cellular level, thus bridging the gap between limitations of assays using either isolated molecules (enzymes, transport proteins) or complex *in vivo* experiments with all the complex feedback mechanisms present. GH3 cell growth, which is measured with the colorimetric AlamarBlue™ assay is increased in the presence of TH agonists and decreased in the presence of TH antagonists.	B - Assessed by the OECD TDM EG. The method identifies chemicals activating the TR, but cannot distinguish THSDCs from e.g. steroids or growth factors. Recommendation for further validation: Identify more active chemicals. Transfer to other laboratories for further validation.	TM2019-17 (EU) ( Ghisari & Bonefeld-Jorgensen, 2005; Ghisari & Bonefeld-Jorgensen, 2009; Gutleb *et al.*, 2005; Kusk *et al.*, 2011; Waring *et al.*, 2012)
NETVAL Method 8b Proliferation, migration and oligodendrocyte maturation (including myelin formation) in mixed neuronal/glial culture (neurospheres) derived from human induced Pluripotent Stem Cells (hiPSC)	TR activation (agonism and antagonism)	THSD can interfere with proliferation, migration and differentiation of oligodendrocytes which may lead to neurodevelopmental impairments. Inhibition or induction of myelination is related to activation or inactivation of TRs. The method encompasses evaluation of these biological processes using mixed neuronal/glial culture derived from human induced Pluripotent Stem Cells (hiPSC) cultured as free-floating neurospheres. Cell viability, proliferation, migration and differentiation measured by expression of myelin specific proteins are assessed following a 1–2-week treatment with test items.	C - This method will be further considered in the context of developmental neurotoxicity by EFSA for which myelination is an important endpoint.	TM2019-18 (EU) ( Chesnut *et al.*, 2021)
NETVAL Method 8c *In vitro* human adipose stromal cell - human umbilical vein endothelial cell (hASC-HUVEC) vasculogenesis/angiogenesis method	TR activation (agonism and antagonism)	THSD can interfere with vasculogenesis and angiogenesis and can be assessed using human primary adipose stromal cells and human primary umbilical vein endothelial cells co-culture without any artificial matrixes. Inhibition or induction of tubule formation is related to activation or inactivation of TRs. In the method the effect on the formation of vasculature is measured six days after the exposure by quantifying the formed tubules using image analysis. Cell viability test (WST-1) is used to measure general cytotoxicity to clarify if the inhibitory mechanism is related to cytotoxicity.	C - The OECD TDM EG is not planning further work on assessment of this method. This method has more relevance for reproductive toxicity (validation status B)	TM2019-19 (EU) ( Toimela *et al.*, 2017)

^3^ A contract research organization

^4^ A contract research organization

### Levels 3 – 5: *In vivo* methods providing data on THSD mechanisms and adverse effects

Assays listed under Levels 3 – 5 are mainly the *in vivo* OECD TGs that were already included in GD 150, including the XETA for which a TG is now available. An important advancement here is the addition of four THSD-sensitive endpoints (TH levels, thyroid morphology/histopathology, impaired swim bladder inflation, altered retinal layer structure through histopathology) to available TGs using fish exposed during development. Fish TGs are part of the standard test requirements in different regulations and inclusion of THSD endpoints in these tests could have immediate regulatory impact. The following augmented TGs have been included in Table 3– Table 5: (1) the THSD Fish Embryo Acute Toxicity (tFET) test (based on TG 236, Level 3, Table 3), (2) the THSD Fish Early Life Stage (tFELS) test (based on TG 210, Level 4, Table 4), and (3) the integrated Fish Endocrine Disruptor Test (iFEDT) (based on merging TGs 229 and 234, Level 5, Table 5). TG 210 is representative of other fish TGs including the early developmental stages like TG 234, TG 240 and the draft Zebrafish Extended One Generation Reproduction Test (ZEOGRT). Table 3– Table 5 further indicate the potential to include additional THSD-relevant endpoints in fish, some of which are being developed in parallel. Examples include the Zebrafish Eleutheroembryo Thyroid Assay (ZETA, NETVAL Method 7a, Table 3) measuring intrafollicular T4 levels, and the Zebrafish Light-dark transition test (LDTT, Table 3) that could be used for assessing THSD-related effects on behaviour ( Atzei *et al.*, 2021; Bilotta *et al.*, 2002; Haigis *et al.*, 2022; Hughes & Hessel, 2024).

**Table 3.** Level 3 methods for THSD evaluation. List of methods for THSD evaluation that fit into Level 3 “ *In vivo* assays providing data about selected endocrine mechanism(s)/pathway(s)” of the OECD Conceptual Framework for Testing and Assessment of Endocrine Disrupting Chemicals ( OECD, 2018a).

Method title	THSD mechanism / effect	Short method description	Test method readiness/ validation status *A - validated (TG)* *B - optimized, ready to be (pre-)validated/ validation ongoing* *C - not validated* *D - validation unsuccessful*	Reference or link
NON-MAMMALIAN ASSAYS				
Amphibian Metamorphosis Assay (AMA) (OECD TG 231)	Altered amphibian metamorphosis	The Amphibian Metamorphosis Assay (AMA) is a screening assay intended to empirically identify substances which may interfere with the normal function of the hypothalamic-pituitary-thyroid (HPT) axis and result in altered amphibian metamorphosis. *Xenopus laevis* tadpoles are raised until stage 51 at which age they are used to initiate the aquatic exposure experiment with a duration of 21 days (protected life stage). Developmental stage, snout-to-vent length and hind limb length are measured.	A - OECD TG 231	( OECD, 2009)
*Xenopus* Eleutheroembryonic Thyroid Assay (XETA) (OECD TG 248)	TR activation (agonism and antagonism) Altered TH levels	*Xenopus laevis* eleutheroembryos are exposed aquatically from stage NF 45 (approx. 7 dpf) to stage NF47 (10 dpf) (unprotected life stage). The test measures the capacity of a chemical to activate or inhibit the transcription of a genetic construct (THb/ZIP-GFP) either by binding to the TR or by modifying the amount of TH available for transcription. The assay is expected to be responsive to all chemicals that interact with TRs, or that lead to either an increase or a decrease in TH levels. However, this assay cannot be linked directly to specific mechanisms but may rather be used for screening purposes.	A - OECD TG 248	( OECD, 2019)
NETVAL Method 7a Measurement of intrafollicular thyroxine (T4) using zebrafish eleutheroembryos Zebrafish Eleutheroembryo Thyroid Assay (ZETA)	Altered TH levels	The Zebrafish Eleutheroembryo Thyroid Assay (ZETA) exposes zebrafish embryos aquatically for 66 hours from 54 hpf (after hatch) until 5 dpf (unprotected life stage). The ZETA is used to detect direct thyroid gland function disruptors in the thyroid of 5 dpf zebrafish embryos. The intrafollicular T4-content (IT4C) is determined by immunofluorescence signal. IT4C is a physiologically relevant integrative endpoint, as any potential disrupting mechanism of the thyroid gland function (e.g. NIS and TPO) would result in increase or decrease of T4 production.	B - C - Assessed by the OECD TDM EG. Recommendation for further validation: Update the SOP and consider to include the T4 measurement in thyroid follicles in other zebrafish embryo methods.	TM2019-16 (EU) ( Raldúa & Babin, 2009; Raldúa *et al.*, 2012; Thienpont *et al.*, 2011; Thienpont *et al.*, 2013)
THSD Fish Embryo Acute Toxicity (tFET) test ( ERGO project)	Altered TH levels, thyroid gland hyperplasia / hypertrophy, altered retinal layer structure, impaired swim bladder inflation	Endpoints are added to the Fish Embryo Acute Toxicity (FET) Test (OECD TG 236 ( OECD, 2013b)) to detect THSD through analysis of TH levels, thyroid follicle morphology (using the zebrafish Tg(tg:mCherry) line or intrafollicular T4 content visualized by immunofluorescence similar to the ZETA), posterior swim bladder inflation, eye histopathology. Zebrafish embryos are exposed aquatically from 0–2 hpf until 5 dpf at 28.5°C. This extension and change of the FET test are required to assess swim bladder inflation. Other fish species such as Medaka, fathead minnow and stickleback can also be used. Additional endpoints can be added at a later time but are at this point not included in the ongoing validation effort, such as TSH levels, gene expression, impaired swimming, the Light-Dark transition test (LDTT) and various endpoints indicative of DNT (e.g., visual and acoustic motor response (VAMR) assay, spontaneous tail coiling (24 hpf), thigmotaxis (anxiety)). Many of these endpoints are under development in PARC. Variations on this test, e.g., with exposure starting at 48 hours post fertilization have also been proposed ( Jaka *et al*., 2023). The addition of THSD endpoints to the existing OECD TG 236 is part of Project 2.64 on the OECD Test Guidelines work plan “Inclusion of thyroid endpoints in OECD fish Test Guidelines” and supported by AOPs developed in Project 1.35 of the OECD AOP development programme “An AOP network for thyroid hormone system disruption in fish”.	B - Validation is ongoing under the auspice of OECD VMG-Eco. Fifteen labs are involved with four fish species (zebrafish, Medaka, fathead minnow, stickleback) and results are expected 2025–2026.	( Gölz *et al.*, 2022; Gölz *et al.*, 2024a; Knapen *et al.*, 2020; Stinckens *et al.*, 2018) Examples of variations and potential future additions: ( Gutsfeld *et al.*, 2024; Jaka *et al.*, 2023)
Zebrafish Light-dark transition test (LDTT)	Impaired swimming, Altered behaviour	Zebrafish embryos are aquatically exposed to the test compound and behavioural assessment is done at 5 dpf (unprotected life stage). The response of zebrafish to a visual stimulus (switch from light to dark and vice versa) is recorded by tracking changes in activity with an automated video tracking system during a sequence of light and dark periods. Normal behaviour is characterised by low basal activity in light and increased activity upon the switch to darkness. Different protocols are being used, including exposure starting from 6 h or from 48 h post fertilization and different durations of light and dark periods. Can be combined with the tFET and/or ZETA.	B - Validation planned Ring tests are ongoing and OECD validation is planned. Addition of the LDTT as a DNT Zebrafish Eleutheroembryo Assay to the DNT *in vitro* Testing Battery (IVB) is under discussion in the OECD DNT Zebrafish Group in communication with the DNT-IVB group. Another effort is ongoing to determine the reproducibility of the DLTT among different laboratories. Different efforts use slightly different protocols and this poses a challenge for validation.	( Atzei *et al.*, 2021; Bilotta *et al.*, 2002; Haigis *et al.*, 2022; Hughes & Hessel, 2024)

**Table 4.** Level 4 methods for THSD evaluation. List of methods for THSD evaluation that fit into Level 4 “ *In vivo* assays providing data on adverse effects on endocrine-relevant endpoints” of the OECD Conceptual Framework for Testing and Assessment of Endocrine Disrupting Chemicals ( OECD, 2018a).

Method title	THSD mechanism / effect	Short method description	Test method readiness/ validation status *A - validated (TG)* *B - optimized, ready to be (pre-)validated/ validation ongoing* *C - not validated* *D - validation unsuccessful*	Reference or link
MAMMALIAN ASSAYS				
Repeated Dose 28-Day Oral Toxicity Study in Rodents (OECD TG 407)	Altered serum TH levels, altered serum TSH levels, thyroid gland hyperplasia / hypertrophy	Young adult animals (rodents) of both sexes are exposed to test material orally (diet, drinking water, gavage) for a period of 28 days and observed for signs of toxicity. The testing method has been updated (2008) to include immune, neurological and endocrine sensitive endpoints. The study is not performed in a life-stage that is most sensitive to endocrine disruption or to the developing nervous or immune system. Results of the study should not be used alone, but in a weight of evidence approach or trigger further in-depth investigation. THSD-relevant measurements: • TH (T3, T4) in serum (optional) • TSH levels in serum (optional) • thyroid gland weight (optional) • thyroid gland histopathology	A - OECD TG 407	( OECD, 2008)
Repeated dose 90-day oral toxicity study in rodents (OECD TG 408)	Altered serum TH levels, altered serum TSH levels, thyroid gland hyperplasia / hypertrophy	Young adult animals (rodents) of both sexes are exposed to test material orally (diet, drinking water, gavage) for a period of 90 days. This study provides information on the possible health hazards likely to arise from repeated exposure over a relatively limited period of time. This study should allow for the identification of chemicals with the potential to cause neurotoxic, endocrine, immunological or reproductive organ effects, which may warrant further in-depth investigation. THSD-relevant measurements: • TH (T3, T4) in serum • TSH levels in serum • thyroid gland weight • thyroid gland histopathology	A - OECD TG 408	( OECD, 2018b)
Repeated Dose 90-day Oral Toxicity Study in Non-Rodents (OECD TG 409)	Altered serum TH levels, altered serum TSH levels, thyroid gland hyperplasia / hypertrophy	The 90-day study in non-rodents (preferred species: dog) provides information on the possible health hazards likely to arise from repeated exposure to test material over a period of rapid growth and into young adulthood. The study should allow for the identification of chemicals with the potential to cause neurotoxic, endocrine, immunological or reproductive organ effects, which may warrant further in-depth investigation. THSD-relevant measurements: • TH (T3, T4) in serum (optional) • TSH levels in serum (optional) • thyroid gland weight • thyroid gland histopathology Note this assay is not in general use. A virtual dog assessment tool is under development ( Yang *et al.*, 2022)	A - OECD TG 409	( OECD, 1998)
Prenatal developmental toxicity study (OECD TG 414)	Altered serum TH levels, altered serum TSH levels, thyroid gland hyperplasia / hypertrophy	Pregnant animals (rodent preferred species: rat; non-rodent preferred species: rabbit) are exposed to test material preferably orally by intubation at least from implantation to one day prior to the day of killing (as close as possible to the normal day of delivery). Assay examines organogenesis, effects from preimplantation through entire period of gestation. THSD-relevant measurements (dams only): • TH (T3, T4) in serum • TSH levels in serum • thyroid gland weight • thyroid gland histopathology	A - OECD TG 414	( OECD, 2018c)
Reproduction/developmental toxicity screening test (OECD TG 421)	Altered serum TH levels, altered serum TSH levels, thyroid gland hyperplasia / hypertrophy	Young adult animals (preferred species: rat) are exposed to test material orally (diet, drinking water, gavage) from premating and mating periods and at least until a minimum total dosing of 28 days in males and 63 days in females (i.e. at least 14 days premating, up to 14 days mating, 22 days gestation, 13 days lactation). Thus, exposure covers some of the sensitive periods during development (pre- or early postnatal periods). This study can be used to provide initial information on possible effects on reproduction and/or development, but it does not provide complete information on all aspects of these processes. THSD-relevant measurements: • T4 levels in serum, total: pups/day 4 (optional), pups/day 13, dams/day 13 (optional), adult males • Other hormones (e.g., TSH) (optional) • Thyroid gland weight (pups, adults), optional • Thyroid gland histopathology (pups, adults), optional	A - OECD TG 421	( OECD, 2016a)
Combined Repeated Dose Toxicity Study with the Reproduction/Developmental Toxicity Screening Test (OECD TG 422)	Altered serum TH levels, altered serum TSH levels, thyroid gland hyperplasia / hypertrophy	This assay combines a reproduction/developmental toxicity screening part (TG 421 using pregnant animals) with a repeated dose toxicity part (TG 407 using non-pregnant animals) resulting in reduced animal use and a better means of discriminating direct effects on reproduction/development from those that are secondary to other (systemic) effects. Young adult animals (preferred species: rat) are exposed orally (diet, drinking water, gavage) from premating and mating periods and at least until a minimum total dosing of 28 days in males and 63 days in females [i.e. at least 14 days premating, (up to) 14 days mating, 22 days gestation, 13 days lactation]. This study provides information on the possible health hazards likely to arise from repeated exposure over a relatively limited period of time covering some of the sensitive periods during development (pre- or early postnatal periods). It further comprises a reproduction/developmental toxicity screening test that can be used to provide initial information on possible effects on male and female reproductive performance such as gonadal function, mating behaviour, conception, development of the conceptus and parturition. Compared to TG 421, the study also places emphasis on neurological effects (sensory activity, grip strength and motor activity assessments) as a specific endpoint as well as haematology or clinical chemistry. THSD-relevant measurements: • T4 and TSH levels in serum: pups/day 4 (optional), pups/day 13-termination, dams/day 13-termination (optional), adult males/ termination • Thyroid gland weight (pups, adults), optional • Thyroid gland histopathology (pups, adults), optional • Animals in a satellite group scheduled for follow-up observations should be kept for a further 14 days after the first scheduled kill of dams to detect delayed occurrence, persistence, or recovery from toxicity.	A - OECD TG 422	( OECD, 2016b)
Carcinogenicity studies (OECD TG 451)	Thyroid gland hyperplasia / hypertrophy	Young adult animals (rodents) are exposed orally (diet, drinking water, gavage), dermally, or by inhalation for a period of 24 months. This assay provides information on the possible health hazards likely to arise from repeated exposure for a period lasting up to the entire lifespan of the species used. THSD-relevant measurements: • Thyroid gland weight • Thyroid gland histopathology	A - OECD TG 451	( OECD, 2018e)
Chronic toxicity studies (OECD TG 452)	Altered serum TH levels, altered serum TSH levels, thyroid gland hyperplasia / hypertrophy	Young adult animals (rodent preferred species: rat; non-rodent preferred species: dog) are exposed orally (diet, drinking water, gavage) for a period of 12 months (although shorter or longer durations may be chosen). This assay provides information on the possible health hazards likely to arise from repeated exposure over a considerable part of the lifespan of the species used. Chemicals indicated in the 28-day and/or 90-day toxicity tests to cause neurotoxic effects may optionally be assessed for sensory reactivity to stimuli of different types (e.g., auditory, visual and proprioceptive stimuli), assessment of grip strength and motor activity. THSD-relevant measurements: • Thyroid gland weight • Thyroid gland histopathology • Specific hormones (optional)	A - OECD TG 452	( OECD, 2018f)
Combined chronic toxicity/carcinogenicity studies (OECD TG 453)	Altered serum TH levels, altered serum TSH levels, thyroid gland hyperplasia / hypertrophy	Young adult animals (rodents) are exposed orally (diet, drinking water, gavage) for a period of 12 months for the chronic toxicity phase of the study (although shorter or longer durations may be chosen) and 18 or 24 months for the carcinogenicity phase of the study depending on species and strain used. This assay aims to identify chronic toxicity in combination with carcinogenicity in animals exposed over the majority of the normal life span. Chemicals indicated in the 28-day and/or 90-day toxicity tests to cause neurotoxic effects may optionally be assessed for sensory reactivity to stimuli of different types (e.g., auditory, visual and proprioceptive stimuli), assessment of grip strength and motor activity. THSD-relevant measurements: • Thyroid gland weight • Thyroid gland histopathology • Specific hormones (optional)	A - OECD TG 453	( OECD, 2018g)
Pubertal Development and thyroid function in intact juvenile / Peripubertal female rats assay (US EPA 890.1450)	Altered serum TH levels, altered TSH levels, thyroid gland hyperplasia / hypertrophy	Juvenile female rats are exposed to test material orally (gavage) from post-natal day (PND) 22 to PND42. This assay is used to detect not only anti-thyroid, estrogenic or anti-estrogenic chemicals, but also agents that alter pubertal development through mechanisms that induce changes in luteinizing hormone, follicle stimulating hormone, prolactin or growth hormone levels or via alterations in hypothalamic function. THSD-relevant measurements: • T4 levels in serum, total • TSH levels in serum • Thyroid gland weight • Thyroid gland histopathology	A - US EPA 890.1450	( USEPA, 2009a)
Pubertal Development and thyroid function in intact juvenile / Peripubertal male rats assay (US EPA 890.1500)	Altered serum TH levels, altered TSH levels, thyroid gland hyperplasia / hypertrophy	Juvenile male rats are exposed to test material orally (gavage) from PND23 to PND53. This assay is used to detect not only anti-thyroid, androgenic, or anti-androgenic chemicals, but also agents that alter pubertal development through mechanisms that induce changes in gonadotropins, prolactin or via alterations in hypothalamic function. THSD-relevant measurements: • T4 levels in serum, total • TSH levels in serum • thyroid gland weight • thyroid gland histopathology	A - US EPA 890.1500	( USEPA, 2009b)
Guidance for Thyroid Assays in Pregnant Animals, Fetuses and Postnatal Animals, and Adult Animals Also referred to as Comparative Thyroid Assay (CTA)	Altered serum TH levels, altered serum TSH levels, thyroid gland hyperplasia / hypertrophy	Pregnant animals (preferred species: rat) are exposed orally by gavage or by the principal route of potential human exposure from GD6 through GD20 and PND21. Direct pup administration by gavage is optional. Assay examines thyroid function of adult female rats during gestation and lactation, and their offspring prenatally and postnatally. This is also referred to as the Comparative Thyroid Assay (CTA). A modified, downsized CTA with additional measurements (TH levels in brain and foetal brain histology) has recently been proposed ( Minami *et al.*, 2023; Minami *et al.*, 2024). THSD-relevant measurements (dams, foetuses, pups): • TH (T3, T4) levels in serum • TSH levels in serum • Thyroid gland weight • Thyroid gland histopathology • TH (T3, T4) levels in foetal serum and brain (modified CTA)	C	( Minami *et al.*, 2023; Minami *et al.*, 2024; USEPA, 2005)
Streamlined Protocol to Determine Organ-Specific Regulations of Deiodinase 1 and Dehalogenase Activities as Readouts of the Hypothalamus-Pituitary-Thyroid-Periphery-Axis (ATHENA project)	DIO1 activity, IYD activity	Measurement of DIO1 and IYD (DEHAL 1) activity by means of the SK reaction in ex vivo samples of hypo- and hyperthyroid mice of two age groups (young; 3 months and old; 20 months). This provides information on the THS status in vivo.	C	( Renko *et al.*, 2022)
Periventricular heterotopia as marker of hypothyroidism (ATHENA project)	THSD-related DNT	Periventricular heterotopia (ectopic neuronal clusters due to disrupted neuronal migration) in rats is proposed as a marker of adverse effects on brain development, caused by low levels of circulating T4 during development ( Ramhoj *et al.*, 2021). In mice, due to the lower incidence and smaller size of ectopic neuronal clusters, the use of this endpoint is not recommended ( Ramhoj *et al.*, 2023b).	C	( Gilbert *et al.*, 2014; O'Shaughnessy *et al.*, 2018; Ramhoj *et al.*, 2021)
Developmental toxicity studies; Changes in cortical maker gene expression and locomotor activity (ATHENA project)	THSD-related DNT	The purpose is to show the link between TPO inhibition, low concentrations of serum T4, changes in cortical gene expression (a battery of 10 previously identified genes) and adverse behavioural effects	C	( Ramhoj *et al.*, 2022)
Mouse in utero, Striatum and serum, Gavage of gestating mother from midgestation. Analysis of pups (ERGO project)	Altered TH levels, THSD-related DNT	Gavage of gestating mother from midgestation (gestation day 7 to post-natal day 14, 30 min). Changes in striatum transcriptome (RNA sequencing) and serum metabolome (mass spectrometry) are analysed in the pups and could reflect an adverse effect on neurodevelopment. These methods were able to detect minor disruptions of TH signalling in vivo.	C	( Poulsen *et al.*, 2024)
BDNF levels	THSD-related DNT	Alteration in BDNF levels is known to be related to THSD. There are several commercial double antibody sandwich ELISA kits that can be used for identification of BDNF levels in human biological fluids (whole blood, plasma, serum) ( Trajkovska *et al.*, 2007). Methodological considerations that have to be taken into account during sample preparation and measurement of BDNF by ELISA have been recently reviewed in Elfving *et al.* 2010., A study measuring BDNF by a commercially available ELISA kit in various tissues and biological liquids derived from distinct species revealed that BDNF is undetectable in mouse blood and pig plasma ( Klein *et al.*, 2011). This study also showed that in most cases BDNF levels are comparable to levels reported in humans and that there is positive correlation between blood BDNF levels and hippocampal BDNF levels in rats and pigs ( Klein *et al.*, 2011).	C	( Elfving *et al.*, 2010; Klein *et al.*, 2011; Trajkovska *et al.*, 2007)
DNA methylation of BDNF	THSD-related DNT	Assessment of DNA methylation of BDNF in whole blood using Bisulphite Pyrosequencing. DNA methylation of the BDNF gene is considered to be a more sensitive marker for DNT than the BDNF protein/gene levels.	C	( Kowiański *et al.*, 2018; Kundakovic *et al.*, 2015)
NON-MAMMALIAN ASSAYS				
THSD Fish Early Life Stage (tFELS) test ( ERGO project)	Altered TH levels, thyroid gland hyperplasia / hypertrophy, altered retinal layer structure, impaired swim bladder inflation	Endpoints are added to the Fish Early Life Stage (FELS) Test (TG 210 ( OECD, 2013a)) to detect THSD through analysis of TH (at least T3, T4) levels, thyroid follicle morphology (by histopathology), posterior and anterior swim bladder inflation and eye histopathology. Fish embryos are exposed from immediately after fertilization until 30 days post hatch. Different fish species (zebrafish, Medaka, fathead minnow and stickleback) can be used. Additional endpoints such as TSH levels, gene expression and impaired swimming can be added at a later time but at this point are not included in the ongoing validation effort.	B - Validation is ongoing under the auspice of OECD VMG-Eco. Fifteen labs are involved with four fish species (zebrafish, Medaka, fathead minnow, stickleback) and results are expected 2025–2026. Addition of THSD endpoints to the existing OECD TG 210 is part of Project 2.64 on the OECD Test Guidelines work plan “Inclusion of thyroid endpoints in OECD fish Test Guidelines” and supported by AOPs developed in Project 1.35 of the OECD AOP development programme “An AOP network for thyroid hormone system disruption in fish”.	( Knapen *et al.*, 2020; Stinckens *et al.*, 2020)
The Larval Amphibian Growth and Development Assay (LAGDA) (OECD TG 241)	Thyroid gland hyperplasia / hypertrophy, altered amphibian metamorphosis	The assay has three endpoints indicating generalised toxicity (mortality, abnormal behaviour and growth), and several providing specific information about endocrine disruption or impaired reproduction (histopathology of thyroid, gonads, kidney and liver, time to metamorphosis [NF stage 62]; secondary sex characteristics (nuptial pads); vitellogenin (optional); genetic and phenotypic sex ratio). Most of these specific endocrine endpoints are likely to respond to interference with the hypothalamic/pituitary/gonadal (HPG) axis, while thyroid histopathology and time to metamorphosis may respond to interference with the hypothalamic/pituitary/thyroid axis (as may the “generalised toxicity” indicator, growth).	A - OECD TG 241	( OECD, 2015)

**Table 5.** Level 5 methods for THSD evaluation. List of methods for THSD evaluation that fit into Level 5 “In vivo assays providing more comprehensive data on adverse effects on endocrine-relevant endpoints over more extensive parts of the life cycle of the organism” of the OECD Conceptual Framework for Testing and Assessment of Endocrine Disrupting Chemicals ( OECD 2018a).

Method title	THSD mechanism / effect	Short method description	Test method readiness/ validation status *A - validated (TG)* *B - optimized, ready to be (pre-)validated/ validation ongoing* *C - not validated* *D - validation unsuccessful*	Reference or link
MAMMALIAN ASSAYS				
Extended one-generation reproductive toxicity study (EOGRTS) (OECD TG 443)	Altered serum TH levels, altered TSH levels, thyroid gland hyperplasia / hypertrophy	This study evaluates the effects of pre- and postnatal chemical exposure on fertility as well as specific developmental life stages of rodents (preferred species: rat), and includes developmental endpoints, reproductive endpoints, as well as specific cohorts on developmental neurotoxicity and developmental immunotoxicity (preferred exposure route: diet). A parental generation, F1 generation and potentially an F2 generation are studied. Developmental neurotoxicity cohort is optionally used for auditory startle, functional observational battery, motor activity and additional neuropathology assessments. Further functional testing (e.g. sensory, social, cognitive) may be added. THSD-relevant measurements: • T4 and TSH levels in serum (PND 22 pups, parents and F1 at termination; optional: T4 in PND 4 pups) • Thyroid gland weight (parents and F1 at termination) • Thyroid histopathology (parents and F1 at termination)	A - OECD TG 443	( OECD, 2018d)
Two-Generation Reproduction Toxicity Study (OECD TG 416)	Thyroid gland hyperplasia/hypertrophy	This study provides general information on effects on the integrity and performance of the animal reproductive systems and growth and development of the offspring in rodents (preferred species: rats). Animals are exposed during pre-mating, mating, pregnancy, weaning, growth and up to weaning for two successive generations (preferred exposure route: oral). THSD-relevant endpoints: Thyroid weight (parents and F1 at termination) and histopathology (optional).	A - OECD TG 416	( OECD, 2001)
NON-MAMMALIAN ASSAYS				
Avian Two-Generation Toxicity Test in the Japanese Quail (ATGT) (US EPA 890.2100)	Altered TH levels, thyroid gland hyperplasia / hypertrophy	This test using Japanese quail is designed to evaluate health and reproductive viability of the first F1 generation following parental exposure (preferred exposure route: dietary). The test covers different life stages: in ovo, juvenile, subadults, and adults. The number of F2 14-day old survivors per F1 generation hen is the primary biological endpoint of this test, although the test includes an option of extending the study through reproductive maturity of the F2 generation. THSD-relevant measurements (parents, offspring): • T4 levels in plasma and thyroid gland • Thyroid gland weight • Thyroid histopathology	D - US EPA 890.2100 Despite efforts to demonstrate the robustness and reproducibility of the test, given the logistical complexity, the numerous sources of possible failure of the test, and the large animal number used in the test to achieve statistical power, countries decided to stop the development of a harmonised OECD Test Guideline in 2014.	( USEPA, 2015; OECD, 2017)
integrated Fish Endocrine Disruptor Test (iFEDT)	Altered TH levels, thyroid gland hyperplasia / hypertrophy, altered retinal layer structure, impaired swim bladder inflation	The iFEDT merges the existing OECD TGs 229 (fish short-term reproduction assay) and 234 (fish sexual development test) that are focused on reproductive and developmental toxicity and implements thyroid-related endpoints like the endpoints that are under validation for addition to TG 236 (tFET test, Table 3) and TG 210 (tFELS test, Table 4). The test covers multiple life stages including reproduction, early development and sexual differentiation, and thus allows the identification of multiple ED modalities in fish. So far, the test has been performed with zebrafish. Parental fish are aquatically exposed for 21 days and offspring is exposed until 60 dph. In addition to the existing reproductive endpoints from TG 229 and 234, THSD endpoints are studied in parental fish, 5 dpf embryos and juveniles.	B - The test has been developed and performed with a limited set of compounds. Validation is foreseen in the near future, but has not been planned yet.	( Fagundes *et al.*, 2024; Gölz *et al.*, 2024b; Pannetier *et al.*, 2024)

Related to mammalian *in vivo* testing, efforts have been made to explore the use of additional endpoints in rodent assays to better characterize the effects on the THS, such as the modified Comparative Thyroid Assay (CTA) with inclusion of TH level measurements in foetal serum and brain ( Minami *et al.*, 2023; Minami *et al.*, 2024; USEPA, 2005) as well as some downstream effects associated with THSD. Effects on brain development and function have received a lot of attention especially, such as the work in the ATHENA project on brain heterotopia (ectopic neuronal clusters due to disrupted neuronal migration) in rats ( Audouze *et al.*, 2024; Ramhoj *et al.*, 2021; Ramhoj *et al.*, 2022) ( Table 4). Another example is the inclusion into rodent *in vivo* studies of brain-derived neurotrophic factor (BDNF) ( Elfving *et al.*, 2010; Klein *et al.*, 2011; Trajkovska *et al.*, 2007) and BDNF methylation ( Kundakovic *et al.*, 2015; Kowiański *et al.*, 2018), an effect biomarker strongly linked to THSD ( Rolaki *et al.*, 2019) ( Table 4).

Many different adverse effects have been associated with THSD and for some of those, important method development efforts are ongoing in parallel, e.g., the developmental neurotoxicity *in vitro* test battery (DNT IVB, Blum *et al.*, 2023). The inclusion of methods addressing any THSD-relevant adverse effect was considered outside the scope of the present effort.

## Concluding remarks

Over the past decade, significant progress has been made with the development and validation of Level 1-3 methods for THSD, with advancements in in silico models, *in vitro* assays (in particular via EU-NETVAL), and methods using non-protected fish embryonic life stages (inclusion of THSD-sensitive endpoints in the FET test). These advances directly address the need for reducing reliance on animal testing in the context of enhancing chemical safety assessments of endocrine disruptors, one of the Key Areas of Regulatory Challenge (KARC) recently identified by ECHA ( ECHA, 2024). Although it is recognized that at this time, it is not possible to move completely away from *in vivo* animal testing for ED, New Approach Methodologies (NAMs), such as in silico models, *in vitro* tests and *in vivo* tests with fish and amphibian embryos, are crucial to improve the hazard assessment of these substances. Once experience has been gained, and validation - demonstrating relevance, reliability and robustness - has been successfully achieved with adequate sensitivity, this will increase confidence in using such NAMs, so that these new methods will be expected to be ready for implementation into regulatory programmes in the medium-term future.

In addition, THSD-sensitive endpoints have been added to the Fish Early Life Stage (FELS) test at Level 4, and a transgenerational fish test incorporating the same THSD-sensitive endpoints (integrated Fish Endocrine Disruptor Test, iFEDT) has been developed at Level 5. Since OECD validation of these fish THSD endpoints in the FET and FELS tests is ongoing and is expected to be finalized around 2026, it is expected that these advances can be implemented into regulatory programmes in the short-to-medium term. For example, while amphibian tests for evaluating THSD are currently available, the EU REACH regulation (EC1907/2006) covering all industrial chemicals does not require amphibian testing, but a number of fish tests, including TG 210, are part of the standard information requirements (dependent on the tonnage of the chemical). Inclusion of the augmented fish TGs therefore has the potential to directly serve both REACH and the hazard classes for ED that have recently been added to the CLP regulation.

This work addresses the international need for a comprehensive reference list of methods that can be used for the evaluation of THSD, noting that it is not fully exhaustive given the ongoing developments in this active field of applied research for regulatory needs. The methods overview provided here offers a state of the methods available for THSD evaluation as of September 2024, and should be viewed as a resource that can be built upon and expanded. The methods presented here are based on mapping and inventory exercises that were carried out within the PARC framework. This list can serve as a point of reference for future developments, validation efforts, and readiness assessments in related ongoing international activities. Importantly, our method inventory highlights the need for updating GD 150 to reflect the most recent advances in THSD method development and validation (amongst other recently adopted endocrine relevant TGs), ensuring that regulatory frameworks remain aligned with the latest scientific test method developments. This work is also relevant within the broader context of the United Nations Globally Harmonized System (UN GHS) for chemical classification where inclusion of endocrine disruptors as a hazard class in the GHS is under discussion for implementation.

## Data Availability

No data are associated with this article. **Zenodo:** Title: “Extended Data supplementary to the publication: A 2024 inventory of test methods relevant to thyroid hormone system disruption for human health and environmental regulatory hazard assessment”. 10.5281/zenodo.13934576. Extended Data consist of (1) a list of additional in silico and *in vitro* methods that study an indirect link with THSD .docx (2) information on a targeted literature search for availability of in silico models for thyroid hormone system disruption. xlsx Data are available under the terms of the Creative Commons Attribution 4.0 International

## Footnotes

^1^ The assessment of the reliability (or reproducibility) and relevance (predictive capacity) of a particular test, approach, method, or process with an associated prediction model established for a specific purpose ( Jacobs *et al.*, 2024; OECD, 2005)
