# Supplementary material for: A 2024 inventory of test methods relevant to thyroid hormone system disruption for human health and environmental regulatory hazard assessment
Source: Open Res Eur. 2024 Nov 6;4:242. [Version 1] doi: 10.12688/openreseurope.18739.1 (PMC11809485; doi:10.12688/openreseurope.18739.1)
Supplement: Peer review history

# Reviewer report: Reviewer response for version 1

**DOI:** 10.21956/openreseurope.20273.r46946

**Published:** 2025-01-27

**Authors:** Jean-Baptiste Fini

**Affiliations:** 1 CNRS UMR 7221, Muséum National d’Histoire Naturelle, Paris, France

Review  “A 2024 inventory of test methods relevant to thyroid hormone system disruption for human health and environmental regulatory hazard assessment

 Summary of the Article

 Vergauwen et al. outline a detailed inventory of test methods applicable to assess thyroid hormone system disruption (THSD). It encompasses 108 methods in the five levels of the OECD Conceptual Framework on in silico, in vitro and in vivo approaches. This manuscript addresses the lack of sufficient validated methods to be used for THSD testing and discusses significant developments on regulatory issues, focusing on IATA’s.

 The authors have done a great job in organizing and analyzing the different techniques, their availability and readiness for regulatory processes as a good working tool for the researchers and regulators.This inventory also emerges as a resource that assists in the formulation of the Integrated Approaches to Testing and Assessment and is in tune with the ongoing efforts of the OECD.

 The authors, all recognized in the fields of risk assessment, test development for regulatory use, and/or disruption of the thyroid axis, provide a comprehensive review of 108 tests validated, under validation, or in development for detecting thyroid hormone system disruption (THSD), presented in table format. The authors, who are all involved in the European PARC project, have updated the validated data from the OECD's GD150 guidance with newly developed tests from EURION cluster projects (ERGO, ATHENA, SCREENED for the thyroid, as well as GOLIATH and OBERON for translatable tools).

 For each test, the tables provide information on the test's origin, a brief description, a "readiness" level, and references. The tests are organized into tables according to the five OECD levels 1) Data already generated (in silico, QSAR) 2) Mode of action using in vitro methods 3) Mode of action in vivo 4) Adversity 5) Adversity and trans-generational effects.

 This way of presenting the data also highlights numerous pre-validation initiatives (e.g., the PEPPER platform), validation efforts (e.g., the OECD Thyroid Subgroup), and refinements in test guidelines through the addition of multiple parameters to better identify thyroid-specific modalities or predict neuro-developmental effects.

 This results in a very coherent and highly relevant body of work, dense, easy to read despite the numerous acronyms. I would add that this review was necessary, eagerly anticipated, and extremely useful at a time when the EURION cluster is concluding, and the US EPA and Japan are proposing new methods to more finely assess thyroid axis disruptions. Indeed, it is essential to take stock of the different methods to capitalize on promising approaches (although not all are ready for validation), identify gaps, and further develop strategies for test batteries.

 I have some comments that could be considered to improve the accessibility and consistency of the various tables.

 General Comments:

 The introduction and discussion sections are somewhat brief for readers who are not familiar with the field. It would be helpful to highlight the fundamental differences between risk assessment for human health (based only on mammals) and for the environment (which includes non-mammals and wild mammals). Additionally, it is worth emphasizing that this binary distinction is not justified by differences between species. Indeed, all vertebrates (and invertebrates) synthesize T4 and T3 (or iodothyronines) through a complex mechanism involving iodine storage, iodine organification, and tyrosine iodination with coupling in an oxidative environment. It is absolutely fascinating to consider that this complexity has not diverged across species.

 It is also important to note that all vertebrates experience a peak in thyroid hormones during development: around birth for mammals or the driver of amphibian metamorphosis. This implicitly explains why amphibians were initially the primary focus for testing thyroid axis disruption in environmental assessments.

 It should be emphasized that while measuring hormone levels or TSH is certainly informative, it is not sufficient to predict in situ effects on various organs or disruption effects at critical moments (note that authors already reiterated this notion, several times in the table).

 It might be helpful to include a diagram illustrating the different levels of thyroid axis disruption. If the authors prefer not to create such a diagram, many examples can be cited (e.g., Gilbert et al., 2020, or Mughal et al., 2018).

 Additionally, for those less familiar with thyroid hormone signaling, it may be challenging to grasp that disruptions of TPO, TTR, DIO1, 2, 3, or NIS are specific to thyroid hormones, whereas disruptions of phase 1 enzymes are less. Similarly, it might be difficult for non-specialists to understand, unless explicitly mentioned, that many level 2 tests using THSD binding to TR alpha or beta receptors are of limited utility, given that the TR receptor has the smallest ligand-binding pocket among nuclear receptors (Gallastegui et al, 2015) *.*

 Readers might question why phase 1 enzyme tests are listed with other molecular disruption events. It can also be hard for non-specialist readers to understand why the level 4 zebrafish mobility test is included. While this parameter is sensitive to thyroid hormones and inhibitors, it is not diagnostic. Note that this motility effect is conserved in amphibians (see Fini et al., 2017; Spirhanzlova et al., 2022; Leemans 2023; or  Couderq et al., 2020, for reviews of test parameters in non-mammalian parameters).

 Moreover, it should be clarified that while a neurotoxic effect can result from thyroid axis disruption, not all developmental neurotoxicity (DNT) effects are necessarily thyroid system-related.

 Minor points:

 Page 5 XETA stands for Xenopus eleuthroembryo thyroid assay

 Page 9 table 2: it could be interesting to cite the dates when these assays were developed

 Page 11 table 2: netval method 2b should be before method 2c.

 Page 13 table 2 : Netval Method 3b was also used in ATHENA project

 Page 16 Table 2: DIO1 method had been selected for prevalidation in PEPPER_ should be mentioned

 Page 16 Table 2 Netval method 4b: I think this method was not endorsed by the OECD Thyroid working group after evaluation. The grade should be changed to D

 Page 18/19: All assays Glucuronidation and sulphation Phase 2 should be mentioned as it is for Phase1

 Page 23: in the table “cellular responses” it seems a bit weird that the first assay listed is a cell-free system

 Page 24: there is no mention of the thyroid explant (Antonica F, et al., 2012 [Ref 1])

 Page 25: for the T screen assay the paper from Shriks M, et al., 2006 (Ref 2)

 Page 25: for the DNT there is no mention of the human neurosphere assays developed by Ellen Fritsche, maturated in ENDPOINTS and in prevalidation at Koch K, et al., 2022 (Ref 3)

 Page 27: Was the AMA part of the ERGO project? If so, this should be mentioned. In the description of this assays it should be harmonized as it is for the methods as described page 29 ie with parameters in bullet points and mention the thyroid gland histology as optional (but highly recommended)

 Page 27: XETA: should be mentioned that this method was used and ameliorated in the ATHENA project. It should also be mentioned that the assay is a screening assay based in which THSD are tested in co exposure with T3 5nM

 Page 27: It is worth mentioning the TETHYS new system_Pesce E, et al., 2024 (Ref 4)

 Page 28: Since the Zebrafish LDTT is not purely TH specific, I wonder if this assay should appear here.

 Page 30: I always wonder about using “pregnant” for non human mammals. Change to gravid?

 Page 34: Developmental toxicology studies: mention rat or mouse models. hould AOP 42 linking TPO and DNT be mentioned in the references?

 Page 34: BDNF levels; AOP 54 should be cited

 Page 35: LAGDA: Explain that exposure starts at NF8 and is all along development (after metamorphosis)

 Page 35 it is worth mentioning the LATT new assay

 Page 37: iFEDT I wonder if the classification should be B/C instead of B

Is the review written in accessible language?

Yes

Are all factual statements correct and adequately supported by citations?

Yes

Are the conclusions drawn appropriate in the context of the current research literature?

Yes

Is the topic of the review discussed comprehensively in the context of the current literature?

Yes

Reviewer Expertise:

Thyroid, endocrine disruption, assay development, assay validation

I confirm that I have read this submission and believe that I have an appropriate level of expertise to confirm that it is of an acceptable scientific standard, however I have significant reservations, as outlined above.

## References

[1] : Generation of functional thyroid from embryonic stem cells. *Nature* .2012;491(7422) : 10.1038/nature11525 66-71 10.1038/nature11525 23051751 PMC3687105

[2] : T-screen to quantify functional potentiating, antagonistic and thyroid hormone-like activities of poly halogenated aromatic hydrocarbons (PHAHs). *Toxicol In Vitro* .2006;20(4) : 10.1016/j.tiv.2005.09.001 490-8 10.1016/j.tiv.2005.09.001 16219445

[3] : Scientific Validation of Human Neurosphere Assays for Developmental Neurotoxicity Evaluation. *Front Toxicol* .2022;4: 10.3389/ftox.2022.816370 816370 10.3389/ftox.2022.816370 35295221 PMC8915868

[4] : Testing the sensitivity of the medaka Transgenic Eleuthero-embryonic THYroid-Specific assay (TETHYS) to different mechanisms of action. *Aquat Toxicol* .2024;276: 10.1016/j.aquatox.2024.107081 107081 10.1016/j.aquatox.2024.107081 39305711

# Reviewer report: Reviewer response for version 1

**DOI:** 10.21956/openreseurope.20273.r46945

**Published:** 2024-12-31

**Authors:** Josef Köhrle

**Affiliations:** 1 Charité-Universitätsmedizin Berlin, Berlin, Germany

The author consortium, funded by the EU Horizon PARC project, presents a comprehensive inventory of currently available test methods for thyroid hormone system disruption (THSD) chemicals. These tests may be applied for hazard assessment of chemicals and endocrine disruptors potentially adversely interfering with the highly conserved thyroid hormones (TH) system (THS) in humans, terrestrial and aquatic life forms. Such an approach is of eminent relevance for human health and protection of our global environment, because the THS is one of the key hormonal regulators of development, growth, metamorphic processes, metabolism, anabolic and catabolic reactions and homeostasis of all vertebrates and their predecessors. The TH structure is identical in all species and the components of the THS, the TH biosynthetic and  metabolic pathways, and receptor-mediated action are highly conserved across the species.  

 Authors collate in their manuscript the currently available methods and assays relevant to THSD in a well-structured manner and systematically along the OECD guidance document. They cover in silico, in vitro and in vivo methods, carefully list these in extensive tables), and discuss their stage of development, (experimental and statistical) validation, analytical and functional endpoints and existing critical gaps on the intended (long) way to an integrated approaches to testing and assessment (IATA) system. The latter then needs to be accepted and applied both by the regulatory authorities and producers on a global level for the highly demanding and challenging task to properly and timely evaluate the continuously growing chemical universe and the main relevant health and environmental impacts of its single components (suggested in the range of >350,000 chemicals) and structurally/functionally related groups.

 The methods compiled here are currently at different stages of development, validation and application by teams in academic, regulatory, contract and pharmaceutical industry laboratories, including various European and OECD regulatory authorities.

**General comments:**

 This network of authors should be recognized for their comprehensive coverage and discussion of the current state of the art, research and knowledge and especially for including the marked scientific advances of the last three decades in this important hormone field. Unfortunately, the latter is not yet reflected and the case in most of the test guidelines used by regulatory authorities to evaluate chemicals. Similarly, this is also regrettably still common practice in the chemical-pharmaceutical industry and classical toxicology communities, who largely use outdated concepts of hazard and risk assessment, which are solely based on histo-morphological changes in the TH-producing gland; in rare cases complemented by measurement of blood concentrations of the prohormone thyroxine and the pituitary hormone TSH, the main regulatory signal for thyroid gland activity.

 Such outdated approaches still fully neglect that the majority of interferences with the THS by chemicals and endocrine disruptors occurs outside of the thyroid gland at the levels of

1. distribution of the hydrophobic TH to the target tissues and cells, including the brain,
2. the transmembrane transport of the charged TH and their metabolites across (phospho-)lipid bilayers of cellular membranes,
3. obligatory intracellular activation and inactivation of TH by the deiodinase selenoenzymes,
4. intracellular conjugation and degradation preceding the elimination of TH metabolites
5. modulation of the function of nuclear and mitochondrial of T3 receptors, which act as ligand modulated transcription factors of T3-regulated genes

T3 is considered and the main and most potent bioactive form of TH, while T4 is a pro-hormone, exclusively synthetized and secreted by the thyroid gland and probably devoid or of inherent activity under regular in vivo conditions.

 More than that, deiodination, metabolism and degradation of TH are frequently dubbed “secondary” and thus mainly irrelevant metabolic events, not to be considered in assessment of potential hazards of chemicals and EDC for the complex THS. Such an approach fully neglects that activation of prohormones and inactivation of pro-hormones and active hormones, the most potent and powerful biomolecules regulating development, metabolism, homeostasis and function of intact life forms, including humans, are equally important and relevant like TH biosynthesis. Both, **lack and excess of hormones** can cause adversity and are relevant for disease manifestation. Thus, aspects of hormone metabolism and degradation cannot be considered “secondary” and irrelevant to an endocrine molecular initiating event (MIE) and mode of action (MOA) of a chemical potentially leading to an adverse outcome (AO).

 Current science indicates that only a minor fraction of EDC and chemicals primarily affect the biosynthetic function of the thyroid gland itself or interfere with the T3-binding to its receptor.

 The majority of (adverse) interferences in the THS occurs locally at the various cellular steps of the “pre-receptor control of T3 ligand availability” to the T3 receptor transcription factor complex. While this mode of EDC action may be particularly pronounced in the THS, it is not at all unique to the THS, but also found for other hormone receptors of the nuclear receptor family whose function is modulated by low-molecular weight hormones (e.g. retinoids, seco-steroids like calcitriol, sex steroids and mineralocorticoids).    

 Thus, also the traditional outdated **EATS** terminology, still predominantly used in regulatory context, authorities and legal texts, is highly questionable as it combines different concepts under one roof: **E**strogens and **A**ndrogens (two groups of more or less sex-related steroid hormones), **T**hyroid (the largest endocrine gland, remarkably without its hormones?) Steroidogenic (a broad variety of biosynthetic processes).

**Detailed comments:**

 The ms is full of highly specialized abbreviation but most of these are not explained. This makes reading and understanding of the text and tables extremely difficult, especially for non-insiders. A detailed and complete list of abbreviations needs to be added.

**Level 1** (existing data and existing or new non-test information) in silico methods for THSD evaluation, listed in Table 1, are at the stage of statistically validated methods. This spectrum covers some but not all steps of TH biosynthesis and TR mediated cellular response to TH.

 Authors might provide a short comment to each of these major paragraphs and tables which relevant steps and EDC targets of the THS are still missing and need to be developed and validated.

 p. 4, 1. text paragraph: t **he authors follow the misconception here that (hepatic) TH metabolism, excretion and clearance are “ *indirectly linked***”. As extensively stated and discussed above, TH metabolism is an inherent part of the THS like TH biosynthesis. Based on the homeostasis concept for hormone function and action this second side of the coin cannot artificially be declared *secondary* or *indirect*.

 No explanation or comment is given what might be the difference between TPO inhibition QSAR 1 and 2 in table 1.

**Level 2**: In vitro methods providing data on THSD mechanisms

 At this level marked progress has been made during the last decade, especially based on the extensive funding of this research and development area by the EU in the last decade, extensively compiled in Tab 2.

 Authors list several available methods for TPO, NIS, TTR, TH conjugation, TR, etc., but fail to comment on their relevant differences, advantages,  or knowledge gaps and requirements.

**Level 3 – 5**: in vivo methods providing data on THSD mechanisms and adverse effects

 This is a highly relevant area, but unfortunately, the numbers of assays in development or use are very low, compared to those on level2. Author compile several (guideline-based) assays (fish, amphibia) and present perspectives on some of the very few available mammalian assays.

 They conclude that the currently developed NAMs may be ready for regulatory purposes in the medium-term future but may not fully replace testing in intact organisms for some chemicals. They state that the regulatory frameworks require continuous alignment with the latest science developments.

**Minor, but relevant points:**

 While authors present their inventory in precise scientific terminology and up to date, a few terms and statements are misleading.

 p.6, Level 1 2. Paragraph: authors might more clearly present the perspectives of the IATA concept and the currently available Level 1 methodologies (in silico, in vitro) in order to avoid the misconceptions or frequently biased attempts to erroneously equate new approach methodologies ***(NAM)*** with the term “ *non-animal methods”.*

 A list of abbreviations would be very useful and should be added! Not all of them are explained in the text. Such an effort would markedly increase accessibility to this complex text peppered with many technical, regulatory and scientific (abbreviated) terms.

 Authors might discuss and cite the recent NETVAL OECD assessment of availability and validation of THSD-related test methods (Ref 1 OECD (2024), Thyroid in vitro methods: assessment reports by the thyroid disruption methods expert group: Reports assessing the validation status of assays from the EU-NETVAL activities, OECD Series on Testing and Assessment, No. 403, OECD Publishing, Paris.

Is the review written in accessible language?

Partly

Are all factual statements correct and adequately supported by citations?

Yes

Are the conclusions drawn appropriate in the context of the current research literature?

Partly

Is the topic of the review discussed comprehensively in the context of the current literature?

Yes

Reviewer Expertise:

Experimental and molecular endocrinology, thyroid hormone system, endocrine disruptors, essential trace elements, assays and analysis of endocrine relevant ligands

I confirm that I have read this submission and believe that I have an appropriate level of expertise to confirm that it is of an acceptable scientific standard, however I have significant reservations, as outlined above.

## References

[1] : Thyroid in vitro methods: assessment reports by the thyroid disruption methods expert group. 10.1787/3786c75f-en 10.1787/3786c75f-en

# Reviewer report: Reviewer response for version 1

**DOI:** 10.21956/openreseurope.20273.r47537

**Published:** 2024-12-17

**Authors:** Andrea Cediel-Ulloa, Caroline Agrillo

**Affiliations:** 1 Uppsala University, Uppsala, Sweden; 2 Organismal biology, Uppsala University Department of Organismal Biology, Uppsala, Uppsala County, Sweden

The article from Vergauwen et al. provides a thorough review of available, rejected, and under-development methods to evaluate thyroid hormone system disruption (THSD). The review provides a comprehensive analysis of the topic. The authors demonstrate a meticulous investigation, evident not only in the extensive list of citations but also in their thoughtful reflection on the methods used for the literature search. The methods are presented in a tiered manner, according to the OECD conceptual Framework, and their relevance, readiness, and validation status are summarized. Notably, the inclusion of failed assays among the listed methods highlights the authors' careful examination of available approaches, offering valuable references for future assay searches. The review is written in clear, accessible language, making it easy to follow and understand.

 Minor comments:

1. There is an inconsistency in the use of italicizations of method use (i.e., in vitro, in vivo, in silico, in chemico, in utero. This is throughout the whole article, including body text and text in tables.
2. On page 2, the start of the words New Approach Methods (keywords) should be capitalized.
3. On page 5, the abbreviation US EPA is repeated twice.
4. On page 8, the abbreviation JRC is mentioned, although already mentioned on page 5.
5. On page 18, the first row of the table it says “refs to be added”. Please remember to add refs.
6. On page 24, second row of the table, it says mice. Should be changed to mouse.
7. On page 38, only 15 out of 21 authors are listed for their contributions

Is the review written in accessible language?

Yes

Are all factual statements correct and adequately supported by citations?

Yes

Are the conclusions drawn appropriate in the context of the current research literature?

Yes

Is the topic of the review discussed comprehensively in the context of the current literature?

Yes

Reviewer Expertise:

in vitro toxicology, environmental toxicology, neurodevelopment, endocrine disruption

We confirm that we have read this submission and believe that we have an appropriate level of expertise to confirm that it is of an acceptable scientific standard.

# Reviewer report: Reviewer response for version 1

**DOI:** 10.21956/openreseurope.20273.r47540

**Published:** 2024-12-12

**Authors:** Pieter Vancamp

**Affiliations:** 1 Nantes Université, Institut National de Recherche pour, Nantes, France

The review by Vergauwen et al. aims to provide a comprehensive overview of current methods, ranging from in silico to in vitro and in vivo, for assessing the thyroid hormone-disrupting potential of substances or mixtures. The study is motivated by the need to evaluate hazard risks to both the environment and human health. The authors have conducted an extensive literature search and presented their findings in clearly organized tables, which effectively serve as a reference point for understanding the information provided by each method and the conclusions that can be drawn from them. The authors also assign an arbitrary score to indicate the validation status of each method, categorizing them as validated, in (pre)validation stages, not yet validated, or invalid. This is a valuable addition, as it provides a snapshot of the current state of method development. The text is well-written and accomplishes its goal of offering a clear and thorough overview of the field. The authors, recognized experts in thyroid hormone (disruption) research, cite relevant literature. Beyond its descriptive scope, the review underscores significant gaps in validated models and highlights the need for further research to improve assessments of thyroid hormone signaling disruption by chemicals. By identifying areas for optimization, they also offer strategic insights for advancing this field in the coming years. Overall, the review represents an excellent effort and a highly valuable contribution to the literature.

**Suggestions for Improvement**

 While the review is already robust, the following comments and suggestions may further enhance its impact:

- Extrapolation to human and environmental contexts: The authors correctly emphasize the conservation of thyroid hormone signaling across vertebrates, making these systems relevant models for assessing thyrotoxic potential. However, not all responses are identical across species. Could the authors discuss methods, or the lack thereof, to evaluate how findings from these models—particularly in vitro and in vivo systems—can be extrapolated to humans or environmental contexts?
- Sex-specific discrepancies in toxic potential: The concluding remarks are concise and clear. However, they might benefit from discussing factors that are often underrepresented in toxicology studies, such as sex-specific differences in toxic responses. Should future research prioritize incorporating such factors, and how might they influence the validation of novel methods?
- Developmental vs. adult testing stages: A notable distinction between non-mammalian and mammalian models is the focus on different life stages: non-mammalian tests predominantly target early developmental stages, whereas mammalian assays often involve adults. This distinction is touched upon but could be elaborated in the concluding remarks. What are the implications of this difference when determining the toxic potential of substances? Should testing frameworks account for these discrepancies? Should there be made a difference in the thyrotoxic effects on early-life and adult life stages?

Minor Remarks

- Pre-Validation Terminology: The phrase "ready to be pre-validated" requires clarification. How does this status differ from "ready to be validated"? A more precise explanation would help avoid ambiguity and improve the interpretability of the scoring system.
- Level 1 Methods: In the discussion of Level 1 methods, it may be worth explicitly noting their additional advantage as alternatives to animal testing, aligning with the principles of the 3Rs, and their suitability for high-throughput screening of compounds at varying concentrations.
- Zebrafish Light-Dark Transition Test: In Table 3, the inclusion of the Zebrafish Light-Dark transition test raises questions. The text does not clearly explain how thyroid hormone signaling is mechanistically linked to this behavior. Could alterations observed in this test also stem from other endocrine-disrupting effects? Clarifying this connection would strengthen the rationale for its inclusion as a thyroid hormone-disruption assay.

By addressing these points, the review could become even more comprehensive and impactful, providing the scientific community with an invaluable resource for advancing thyroid hormone-disruption research.

Is the review written in accessible language?

Yes

Are all factual statements correct and adequately supported by citations?

Yes

Are the conclusions drawn appropriate in the context of the current research literature?

Yes

Is the topic of the review discussed comprehensively in the context of the current literature?

Yes

Reviewer Expertise:

Cellular thyroid hormone action, MCT8, in vivo models, neurodevelopment

I confirm that I have read this submission and believe that I have an appropriate level of expertise to confirm that it is of an acceptable scientific standard.
